# Research Progress on the Role of Traditional Chinese Medicine in Regulating Ferroptosis in Cardiovascular Diseases

**DOI:** 10.3390/biology15110824

**Published:** 2026-05-23

**Authors:** Pan Li, Zi-Meng Qi, Shi-Chang Li, Jin-Ling Liang, Tian-Yang Xu, Peng Yu

**Affiliations:** 1 School of Pharmaceutical Sciences, Changchun University of Chinese Medicine, Jilin 130117, China; 25104800147@ccucm.edu.cn (P.L.); 25204897138@ccucm.edu.cn (Z.-M.Q.); 24104800128@ccucm.edu.cn (S.-C.L.); 21103080001@stu.ccucm.edu.cn (J.-L.L.); 2 Innovation Practice Center, Changchun University of Chinese Medicine, Jilin 130117, China; 3 Innovation and Entrepreneurship College, Changchun University of Chinese Medicine, Jilin 130117, China

**Keywords:** cardiovascular diseases, ferroptosis, traditional Chinese medicine, research progress

## Abstract

Ferroptosis is an iron-dependent form of regulated cell death driven by lipid peroxidation, playing a critical role in the pathogenesis of various cardiomyopathies, including hypertrophic, dilated, diabetic, ischemic, doxorubicin-induced, and septic cardiomyopathy, as well as myocardial ischemia–reperfusion injury. This article provides a comprehensive narrative review of the molecular mechanisms of ferroptosis—centered on dysregulation of the GPX4/System Xc^−^ axis, iron metabolism, and lipid metabolism—and its role in cardiovascular diseases, with a specific focus on the cardioprotective effects of Traditional Chinese Medicine (TCM). Through a systematic analysis of recent literature, we highlight active components (e.g., baicalin, ginsenoside Rg3, resveratrol, tanshinone IIA), compound formulations (e.g., Qishen Granule, Zhilong Huoxue Tongyu Capsule), and electroacupuncture therapy, which exert effects via multi-target regulation of ferroptosis-related pathways such as Nrf2/HO-1/GPX4, p53/SLC7A11, and PI3K/AKT. Evidence indicates that TCM interventions effectively alleviate cardiomyocyte ferroptosis by activating the Nrf2 antioxidant pathway to upregulate GPX4/SLC7A11, modulating iron metabolism to reduce labile iron pools, and inhibiting ACSL4/ALOX15-mediated lipid peroxidation, with these effects validated in diverse cardiovascular disease models showing improved cardiac function. Targeting ferroptosis offers a novel therapeutic strategy for cardiovascular diseases, and TCM—with its synergistic multi-component, multi-target, multi-pathway advantages—holds significant potential in this context. Future research should prioritize elucidating complex network mechanisms and advancing clinical translation via high-quality studies to provide new theoretical foundations and drug candidates for cardiovascular disease management.

## Simple Summary

Heart disease remains a leading cause of death globally, and current treatments often struggle to stop the damage from worsening, especially in conditions where the heart muscle becomes thickened or is harmed by chemotherapy drugs. Scientists have recently discovered that a specific type of cell death, driven by iron buildup and fat molecule damage inside heart cells, plays a major role in this process. This review article explores how Traditional Chinese Medicine, which uses natural herb combinations and therapies like acupuncture, could offer a powerful solution. Unlike standard drugs that usually target only one problem, these traditional treatments work through multiple pathways at once. They help the heart by boosting its internal defense system against cell damage, balancing iron levels, and preventing the fatal breakdown of fat molecules. Laboratory and animal studies show that these methods successfully protect the heart from this iron-driven damage. By combining ancient medical wisdom with modern science, this research highlights a promising new approach to treating heart disease. It provides a foundation for developing safer and more effective therapies that could improve patient outcomes and address the urgent need for better treatments that actually slow down the disease itself, not just manage symptoms.

## 1. Introduction

Cardiovascular diseases, a group of myocardial disorders caused by abnormalities in cardiac mechanical and/or electrophysiological function, are etiologically classified into primary and secondary forms according to WHO and AHA systems [1,2,3]. Primary cardiomyopathy, defined as a disease directly targeting the myocardium, encompasses conditions where intrinsic pathology leads to cardiac dysfunction with specific or unknown etiology [4] and is subclassified into genetic (e.g., Hypertrophic Cardiomyopathy (HCM), certain Dilated cardiomyopathy (DCM)), acquired (e.g., stress-induced), and mixed (e.g., DCM) forms [5], characterized by autonomous pathology such as ventricular hypertrophy/diastolic dysfunction in HCM or dilation/systolic impairment in DCM [6]. In contrast, secondary cardiomyopathy arises from myocardial involvement secondary to systemic diseases or external factors (e.g., ischemia, metabolic disorders, infections) [2,7], as seen in ischemic, diabetic, or drug-induced injury, where myocardial damage constitutes part of a broader pathophysiological process. The rising global incidence (≈1:2500) poses significant challenges, as these disorders are major causes of heart failure and sudden cardiac death. Despite advances in revascularization, device therapy (e.g., CRT), and targeted drugs (e.g., beta-blockers, SGLT2 inhibitors), critical limitations persist: primary cardiomyopathies (e.g., HCM) show poor prognosis due to complex genotype–phenotype correlations and progressive fibrosis despite early diagnosis, while treatments for secondary forms (e.g., Diabetic cardiomyopathy (DbCM)) offer only symptomatic relief without modifying disease progression. These unmet needs underscore the necessity of investigating novel mechanisms and developing targeted therapies.

Ferroptosis, an iron-dependent form of regulated cell death driven by lipid peroxidation, has been increasingly implicated in the pathogenesis and progression of various cardiovascular diseases. Its pathological hallmarks include mitochondrial structural abnormalities, accumulation of lipid peroxides, and dysregulation of the antioxidant defense system [8,9]. In multiple cardiomyopathy subtypes—such as ischemia–reperfusion injury-induced cardiomyopathy, diabetic cardiomyopathy, and drug-induced cardiotoxicity models (e.g., Doxorubicin (DOX))—distinct ferroptotic cell death has been observed in cardiomyocytes [10,11,12,13]. This process is tightly regulated at multiple levels, including the System Xc^−^–GSH–GPX4 axis, the iron metabolism network, and lipid peroxidation pathways. Notably, inhibition of ferroptosis (e.g., using specific inhibitors such as Ferrostatin-1) has been shown to significantly attenuate cardiomyocyte injury and improve cardiac function and remodeling [10], suggesting that targeting the ferroptotic signaling pathway may offer novel interventional strategies and therapeutic targets for cardiomyopathy.

Traditional Chinese herbal medicine, with its multi-component and multi-target characteristics, exhibits unique advantages in modulating ferroptosis. Studies have revealed that active compounds—such as flavonoids, saponins, polyphenols, polysaccharides, terpenoids, aldehydes, and lignans—can suppress lipid peroxidation and restore redox homeostasis by regulating key molecules including GPX4, iron metabolism-related proteins (e.g., TFR1, FTH1), and lipoxygenases (LOXs). For instance, isoliquiritigenin enhances antioxidant capacity via activation of the Nrf2/SLC7A11 pathway, while compound formulations such as Qishen Granule improve myocardial microcirculation through synergistic modulation of ferroptosis and inflammatory responses. Additionally, acupuncture and other characteristic therapies of TCM further expand the interventional dimension of herbal medicine by regulating the Nrf2/HO-1 signaling axis.

This review summarizes the molecular mechanisms of ferroptosis and its pathological role in cardiovascular diseases and highlights recent advances in the use of Chinese herbal medicines to inhibit ferroptosis via regulation of iron metabolism, antioxidant activity, and lipid metabolism pathways. The aim is to provide a theoretical foundation and innovative directions for the prevention and treatment of cardiac diseases.

This review aims to provide a comprehensive narrative synthesis of the latest advances regarding the intervention of cardiomyopathy progression by TCM through the regulation of ferroptosis. We strive to delineate the developmental trajectory, core controversies, and future directions of this field by extensively retrieving relevant literature and incorporating representative key studies. However, it is necessary to clarify that this paper follows the methodological framework of a narrative review rather than a systematic meta-analysis. Consequently, no strict inclusion and exclusion criteria were predefined, nor were standardized quantitative procedures implemented for search strategies, data extraction, or risk-of-bias assessment. Although we have endeavored to cover all pertinent literature, this non-systematic approach is susceptible to selection bias and does not provide statistically pooled effect sizes. Readers should fully consider the inherent limitations in the strength of evidence synthesis when interpreting the conclusions of this narrative review.

## 2. Molecular Mechanisms of Ferroptosis and Its Regulatory Network in Cardiovascular Diseases

Ferroptosis, a novel form of regulated cell death first proposed by Dixon et al. in 2012, is characterized by iron-dependent accumulation of lipid peroxides, ultimately leading to disruption of cell membrane integrity and loss of cellular function [14]. This process is intricately regulated by a multidimensional molecular network involving iron metabolism, lipid metabolism, and redox homeostasis [8]. In cardiovascular diseases, aberrant activation of ferroptosis exacerbates cardiomyocyte death, promotes inflammatory responses, and contributes to ventricular remodeling, thereby serving as a critical pathological driver of disease progression [15,16].

### 2.1. Core Regulatory Pathways of Ferroptosis

**Figure 1:**
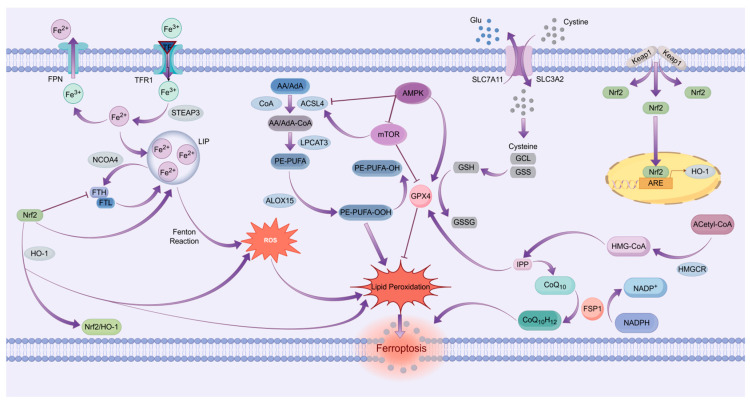
Mechanism of ferroptosis signaling pathway. Ferroptosis is an iron-dependent form of regulated cell death driven by lipid peroxidation. The diagram depicts six core regulatory modules: iron metabolism, lipid peroxidation cascade, System Xc-/GSH/GPX4 axis, Nrf2 antioxidant defense, FSP1/CoQ10/NADPH-independent system, and energy sensing-mediated regulation. (1) Iron metabolism pathway: Transferrin receptor 1 (TFR1) mediates ferric iron (Fe3+) endocytosis, which is reduced to ferrous iron (Fe^2+^) by STEAP3 and enters the labile iron pool (LIP). Ferritin (FTH/FTL) stores intracellular iron; NCOA4-mediated ferritinophagy releases iron. Ferroportin (FPN) exports iron. Fe^2+^ in the LIP catalyzes ROS generation via the Fenton reaction, driving lipid peroxidation. (2) Lipid peroxidation cascade: ACSL4 and LPCAT3 catalyze the esterification of AA and AdA into PE-PUFAs. ALOX15 oxidizes PE-PUFAs to PE-PUFA-OOH, the key executioner molecules of ferroptosis. GPX4 utilizes GSH to reduce toxic lipid hydroperoxides (PE-PUFA-OOH) to harmless PE-PUFA-OH, thereby suppressing ferroptosis. (3) System Xc-/GSH/GPX4 axis: The SLC7A11/SLC3A2 transporter (System Xc-) imports cystine and exports glutamate. Cystine is reduced to cysteine for GSH biosynthesis. GSH is the essential cofactor for GPX4 anti-peroxidative activity. (4) Nrf2 antioxidant defense pathway: Nrf2 is maintained at low levels via Keap1-mediated ubiquitination. Under oxidative stress, Nrf2 translocates to the nucleus, binds ARE elements and activates transcription of target genes including HO-1, conferring resistance to ferroptosis by attenuating lipid peroxidation. (5) FSP1/CoQ10/NADPH-independent antioxidant system: FSP1 utilizes NADPH to reduce CoQ10 to CoQ10H2, directly scavenging lipid radicals as a GPX4-independent parallel protective pathway. IPP participates in CoQ10 synthesis; HMGCR is involved in MVA pathway metabolism. (6) Energy sensing-mediated regulation: AMPK and mTOR sense cellular energy status to positively and negatively regulate ACSL4 activity, respectively, thereby modulating ferroptosis sensitivity. Legend: → (Solid arrow): Activation/Promotion. Indicates that the upstream molecule promotes or activates the function of the downstream molecule. ├ (T-bar): Inhibition. Indicates that the upstream molecule inhibits or negatively regulates the downstream molecule.

The molecular regulatory network of ferroptosis can be conceptualized as a dynamic equilibrium system between “pro-death” and “anti-death” signaling, with its core regulatory nodes comprising the following four key pathways (Figure 1):

#### 2.1.1. The GPX4/System Xc^−^ Axis: The “Scavenger” and Rate-Limiting Switch of Lipid Peroxidation

GPX4 serves as the core inhibitor of ferroptosis, utilizing reduced glutathione (GSH) to reduce lipid peroxides (LPO) into non-toxic lipid alcohols (LOH) and converting hydrogen peroxide (H_2_O_2_) into water, thereby blocking the cascade of lipid peroxidation [14,17,18,19]. System Xc^−^, a transmembrane transporter composed of SLC7A11 and SLC3A2, mediates the 1:1 exchange of extracellular cystine for intracellular glutamate, providing the essential precursor for GSH synthesis (GSH is a tripeptide composed of glutamate, cysteine, and glycine) [20,21]. When System Xc^−^ function is impaired (e.g., via inhibition or gene knockout), cystine uptake is restricted, leading to reduced GSH synthesis. Consequently, GPX4 becomes inactive due to cofactor deficiency, resulting in the accumulation of lipid peroxides and ultimately triggering ferroptosis [22,23]. Thus, the expression level and activity of GPX4, together with the transport efficiency of System Xc^−^, function as critical “switches” governing ferroptosis.

#### 2.1.2. Iron Metabolism Regulation: The “Double-Edged Sword” Effect of Free Iron

Iron serves as an essential catalyst for lipid peroxidation reactions, generating highly toxic hydroxyl radicals (·OH) from H_2_O_2_ via the Fenton reaction. Intracellular iron homeostasis is tightly regulated by an “uptake-storage-export” network: transferrin (Tf) binds to transferrin receptor 1 (TFR1) on the cell membrane to facilitate iron uptake; iron is stored as ferrous ions (Fe^2+^) within ferritin (composed of heavy chain FTH1 and light chain FTL) or exported extracellularly via ferroportin. Nuclear receptor coactivator 4 (NCOA4)-mediated ferritinophagy represents a critical pathway for iron release—NCOA4 binds to ferritin, forming a complex that targets ferritin for lysosomal degradation, thereby releasing free iron [24,25]. Dysregulation of iron metabolism (e.g., TFR1 overexpression enhancing iron uptake, FTH1 downregulation reducing storage capacity, or NCOA4 overactivation accelerating iron release) leads to abnormal accumulation of intracellular free iron, which significantly exacerbates lipid peroxidation and acts as “fuel” for ferroptosis.

#### 2.1.3. Lipid Metabolism-Related Enzymes: The “Synthesis Factory” for Lipid Peroxidation Substrates

The substrates for lipid peroxidation are unsaturated fatty acids (UFA), particularly polyunsaturated fatty acids (PUFA). Acyl-CoA synthetase long-chain family member 4 (ACSL4) specifically catalyzes the binding of PUFAs—such as arachidonic acid (AA) and adrenic acid (AdA)—to coenzyme A (CoA), generating PUFA-CoA. Subsequently, lysophosphatidylcholine acyltransferase 3 (LPCAT3) incorporates PUFA-CoA into phospholipids, forming PUFA-containing phospholipids (PUFA-PL) [26,27]. PUFA-PL serves as the primary substrate for lipid peroxidation, and its elevated levels directly increase the risk of lipid peroxidation. Additionally, lipoxygenases (LOXs, such as 5-LOX, 12-LOX, and 15-LOX) catalyze the non-enzymatic or enzymatic peroxidation of PUFA-PL to generate lipid hydroperoxides (LOOH), while GPX4 terminates this process by reducing LOOH. Therefore, the coordinated action of ACSL4, LPCAT3, and LOXs determines both the “substrate supply” and “reaction rate” of lipid peroxidation.

#### 2.1.4. Other Regulatory Pathways: The “Backup Switches” of the Antioxidant System

Beyond the core pathways mentioned above, ferroptosis is also modulated by multiple interconnected antioxidants signaling pathways:

Nrf2/ARE Pathway: Nuclear factor erythroid 2-related factor 2 (Nrf2), a central transcription factor in cellular antioxidant response, inhibits ferroptosis by activating target genes such as heme oxygenase-1 (HO-1) and NAD(P)H quinone dehydrogenase 1 (NQO1). This activation promotes GSH synthesis, enhances ROS scavenging, and mitigates iron metabolism dysregulation.

AMPK/mTOR Pathway: The energy-sensing kinase AMPK suppresses ferroptosis through dual mechanisms. It phosphorylates and inhibits mTOR, thereby activating autophagy (e.g., clearance of damaged mitochondria) to reduce mitochondrial ROS production. Concurrently, AMPK directly phosphorylates ACSL4, attenuating its activity and decreasing PUFA-PL synthesis.

FSP1/CoQ10 Pathway: Ferroptosis suppressor protein 1 (FSP1), localized to the mitochondrial outer membrane, catalyzes the reduction of coenzyme Q10 (CoQ10) to ubiquinol (CoQH2). As a lipophilic radical-trapping antioxidant, CoQH2 directly neutralizes lipid peroxyl radicals (LOO·), exerting anti-ferroptotic activity independently of the GPX4 system [28]. FSP1 activity thus inhibits lipid peroxide generation and protects against ferroptosis [29].

### 2.2. Pathological Role of Ferroptosis in Cardiovascular Diseases

As highly energy-dependent cells, cardiomyocytes exhibit heightened susceptibility to oxidative stress and dysregulated iron homeostasis. Aberrant activation of ferroptosis contributes significantly to the progression of cardiovascular diseases through the following mechanisms:

#### 2.2.1. Hypertrophic Cardiomyopathy and Dilated Cardiomyopathy

Hypertrophic cardiomyopathy (HCM), the most common primary cardiomyopathy (prevalence ~1:500) [30], is a monogenic heterogeneous disorder caused by over 1400 mutations in more than 11 sarcomeric protein genes [31,32,33,34]. Pathologically, HCM is characterized by enhanced L-type calcium channel (LTCC) activity [35], leading to intracellular calcium overload and an increased risk of arrhythmic sudden death [36,37]. Studies have revealed that LTCC activation also promotes the uptake of non-transferrin-bound iron (NTBI), resulting in intracellular Fe^2+^ accumulation [38,39]. This exacerbates ROS production via the Fenton reaction and drives lipid peroxidation, ultimately triggering ferroptosis. Furthermore, metabolic reprogramming in HCM (e.g., pyruvate/lactate accumulation) [40,41,42,43] and dysregulation of the SLC7A11-p53 pathway synergistically promote ferroptosis [44,45,46]. Animal studies have demonstrated that aortic banding upregulates cardiac NOX4 expression and suppresses GPX4 activity [47,48], while SLC7A11 deficiency exacerbates angiotensin II-induced cardiac hypertrophy and fibrosis [49]. Conversely, the peptide elabela has been shown to improve cardiac function by suppressing ferroptosis, and targeting the ferritinophagy regulator NCOA4 alleviates cardiac iron overload and oxidative damage [50]. Finally, studies have demonstrated that NCOA4 deficiency in murine hearts attenuates left ventricular dilation and restores cardiac function, concurrently reducing ferritin degradation mediated by ferritinophagy following pressure overload. Moreover, hearts lacking NCOA4 exhibit suppressed accumulation of free Fe^2+^ and attenuated overproduction of ROS [51]. Collectively, these findings highlight the central role of ferroptosis in HCM progression.

**Figure 2:**
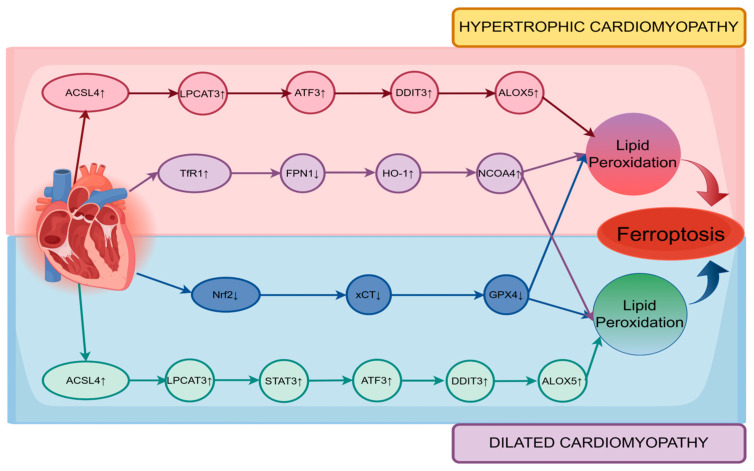
The characteristics of ferroptosis in Hypertrophic Cardiomyopathy and Dilated Cardiomyopathy. This figure illustrates the molecular regulatory mechanisms of ferroptosis in hypertrophic cardiomyopathy (HCM, red-shaded upper panel) and dilated cardiomyopathy (Blue-shaded lower panel). In HCM, ferroptosis is promoted through two distinct pathways. The first pathway involves upregulation of the transferrin receptor (TFR1), downregulation of ferroportin-1 (FPN1), and upregulation of heme oxygenase-1 (HO-1) and nuclear receptor coactivator 4 (NCOA4), collectively leading to iron overload and subsequent lipid peroxidation. The second pathway is characterized by sequential upregulation of acyl-CoA synthetase long-chain family member 4 (ACSL4), lysophosphatidylcholine acyltransferase 3 (LPCAT3), activating transcription factor 3 (ATF3), DNA-damage-inducible transcript 3 (DDIT3), and arachidonate lipoxygenase 5 (ALOX5), which directly drive lipid peroxidation. In DCM, impairment of the antioxidant defense system is mediated by downregulation of nuclear factor erythroid 2-related factor 2 (Nrf2), the cystine/glutamate antiporter system xCT, and glutathione peroxidase 4 (GPX4). Concomitantly, upregulation of ACSL4, LPCAT3, signal transducer and activator of transcription 3 (STAT3), ATF3, DDIT3, and ALOX5 promotes lipid peroxidation, ultimately culminating in cardiomyocyte ferroptosis. Legend: → (Solid arrow): Activation/Promotion. Indicates that the upstream molecule promotes or activates the function of the downstream molecule.

Dilated cardiomyopathy (DCM), the second most common non-ischemic cardiomyopathy with a prevalence of approximately 1:2500 [30], is characterized by severe heart failure symptoms and susceptibility to sudden cardiac death, often requiring heart transplantation as the sole therapeutic option [52]. The pathogenesis of DCM can be classified into genetic and secondary forms. Hereditary DCM accounts for only 20–30% of cases, while the majority are secondary to primary cardiac disorders such as ischemic heart disease, DbCM, and septic cardiomyopathy (SCM) [53,54]. The mechanisms promoting ferroptosis in hereditary DCM share similarities with those in HCM [55]. The figure schematically illustrates the ferroptosis-related pathways implicated in both hypertrophic and dilated cardiomyopathy (Figure 2).

#### 2.2.2. Diabetic Cardiomyopathy

In 2021, the global incidence of diabetes reached 529 million people, and this number is projected to rise to 1.31 billion by 2050 [56]. Diabetes poses a major medical challenge worldwide [57], and diabetic cardiomyopathy (DbCM) is one of its most common complications, as well as a leading cause of heart failure and mortality [58,59,60]. DbCM is characterized by diabetes-induced myocardial injury, presenting with interstitial fibrosis and diastolic dysfunction [61,62]. Pathological features of DbCM include cardiomyocyte hypertrophy, cell death, and fibrosis, with complex underlying mechanisms involving lipid deposition, oxidative stress, hyperglycemia, inflammation, insulin resistance, endoplasmic reticulum stress (ERS), autophagy, and accumulation of advanced glycation end products [63]. Despite extensive research over recent decades, the understanding of DbCM pathogenesis and diagnostic criteria remains limited. Excessive iron accumulation beyond physiological levels can lead to structural myocardial damage and cardiac dysfunction [64] (Figure 2). Recent studies suggest that ferroptosis may play a role in the pathogenesis of DbCM. Iron overload increases the risk of insulin resistance and contributes to cardiovascular disease [65,66], while reducing oxidative stress or enhancing endogenous antioxidant defense systems may effectively treat DbCM [67,68,69]. Therefore, inhibiting iron overload-induced lipid peroxidation could be crucial for enhancing antioxidant capacity and mitigating pathological damage in DbCM [70].

**Figure 3:**
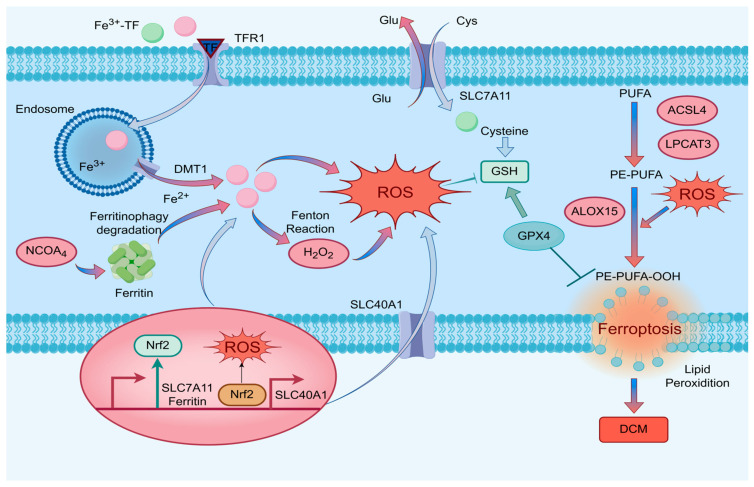
The characteristics of ferroptosis in Diabetic cardiomyopathy. This figure delineates the complete molecular mechanism of ferroptosis signaling pathways in diabetic cardiomyopathy (DbCM). Ferric iron (Fe^3+^) enters the cell via transferrin (TF)-bound endocytosis mediated by the TFR1 receptor. Within the endosome, divalent metal transporter 1 (DMT1) mediates the transport and release of ferrous iron (Fe^2+^). Concurrently, NCOA4-mediated ferritinophagy degrades ferritin, releasing additional Fe^2+^ into the labile iron pool. Free Fe^2+^ catalyzes hydrogen peroxide (H_2_O_2_) decomposition via the Fenton reaction, generating reactive oxygen species (ROS). At the plasma membrane, solute carrier family 7-member 11 (SLC7A11) mediates cystine uptake, which is essential for glutathione (GSH) biosynthesis; GSH serves as the obligate cofactor for GPX4 antioxidant activity. On the lipid metabolism axis, ACSL4 and LPCAT3 facilitate the esterification of polyunsaturated fatty acids (PUFAs) into phosphatidylethanolamine-conjugated PUFAs (PE-PUFAs), which are subsequently oxidized by arachidonate 15-lipoxygenase (ALOX15) to generate PE-PUFA hydroperoxides (PE-PUFA-OOH), a process suppressed by GPX4. When iron overload overwhelms antioxidant defenses, accumulated ROS drive extensive lipid peroxidation, ultimately triggering cardiomyocyte ferroptosis and contributing to DCM pathogenesis. Legend: → (Solid arrow): Activation/Promotion. Indicates that the upstream molecule promotes or activates the function of the downstream molecule. ├ (T-bar): Inhibition. Indicates that the upstream molecule inhibits or negatively regulates the downstream molecule.

Accumulating evidence indicates that dysregulated iron metabolism in diabetes promotes ROS generation, inducing myocardial oxidative stress and cardiomyocyte injury [71,72]. Studies demonstrate that the ferroptosis inducer erastin triggers ferroptosis by accelerating free radical accumulation, and erastin treatment in DbCM models significantly elevates intracellular ROS levels in cardiomyocytes [73,74]. In vitro experiments confirm that ferroptosis inhibitors effectively suppress apoptosis in H9C2 cells. In streptozotocin (STZ)-induced diabetic rats, increased myocardial oxidative stress and reduced left ventricular ejection fraction were observed; however, supplementation with vitamin E (a ferroptosis inhibitor) attenuated oxidative stress and improved hemodynamics, supporting the protective potential of ferroptosis suppression [75,76]. Wu et al. reported enhanced ferroptosis in high-fat diet/STZ-induced diabetic mice [77], mechanistically linked to downregulated SLC7A11 expression, GSH depletion, and exacerbated lipid peroxidation—findings validated in palmitic acid/high glucose (HG)-treated H9C2 and primary cardiomyocytes. Ghosh et al. further documented reduced GSH and elevated ROS in STZ-diabetic rat hearts, promoting cardiomyocyte apoptosis [78]; sulforaphane (SFN) counteracted ferroptosis by activating the AMPK pathway and upregulating ferritin and SLC7A11 expression, ameliorating diabetic cardiac remodeling [79]. The Nrf2/ARE pathway plays a central role in modulating ferroptosis [80]: Nrf2 activation elevates ferritin and SLC7A11 levels to suppress ferroptosis, while Nrf2-mediated SLC40A1 upregulation facilitates Fe^2+^ efflux, reducing ROS production [81,82]. In STZ-diabetic rats, exogenous spermidine attenuated ERS and oxidative stress by inhibiting the Nrf2-ROS-p53-MuRF1 axis, enhancing ROS clearance and calcium-sensing receptor levels, thereby improving cardiac function. Conversely, global Nrf2 knockout exacerbated cardiac remodeling, oxidative stress, and apoptosis, accelerating cardiac dysfunction in type 1 diabetes mellitus (T1DM) [83,84]. Additionally, HO-1 expression was elevated in DbCM, and its knockdown alleviated ferroptosis, cardiac fibrosis, and dysfunction [85]. In summary, ferroptosis contributes to DCM pathogenesis via lipotoxic stress, oxidative damage, and multi-pathway crosstalk, highlighting its potential as a therapeutic target for DbCM intervention (Figure 3).

#### 2.2.3. Ischemic Cardiomyopathy and Myocardial Ischemia–Reperfusion Injury

Ischemic Cardiomyopathy (ICM), primarily caused by chronic ischemia/hypoxia resulting from coronary atherosclerosis, impairs cardiac systolic or diastolic function and represents a major etiology of heart failure (HF) worldwide [86,87]. Additionally, this pathology triggers multiple phenotypic alterations, such as myocardial remodeling and heart failure. During the progression of ICM, reperfusion injury acts synergistically with chronic ischemia through mechanisms including microvascular dysfunction and cardiomyocyte death, collectively driving ventricular remodeling and cardiac dysfunction [88]. During Myocardial Ischemia–Reperfusion Injury (MI/RI) events, inadequate coronary blood supply relative to myocardial demand leads to metabolic disturbances in cardiomyocytes and cell death, subsequently inducing structural and functional cardiac changes [89,90,91]. Reperfusion-associated oxidative damage following IR is closely linked to lipid peroxidation and elevated intracellular iron levels [92]. Current research on the role of ferroptosis in IR-induced myocardial injury predominantly focuses on ERS, reactive oxygen species generation, and the GPX4- and autophagy-dependent ferroptosis pathways [84].

**Figure 4:**
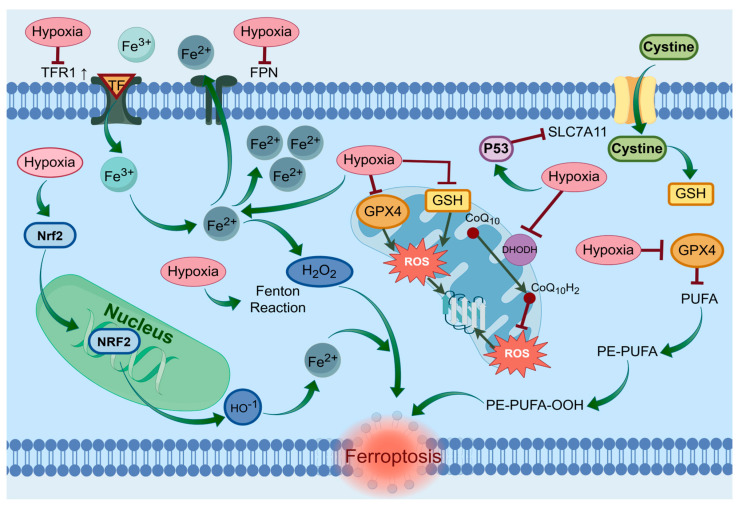
The characteristics of ferroptosis in Ischemic Cardiomyopathy and Myocardial Ischemia–Reperfusion Injury. This figure systematically elucidates the molecular regulatory network of ferroptosis under hypoxic conditions. At the iron metabolism level, hypoxia promotes TFR1 upregulation to enhance iron uptake while suppressing FPN expression to reduce iron efflux, resulting in intracellular Fe^2+^ accumulation; Fe^2+^ subsequently catalyzes ROS generation through the Fenton reaction. Regarding antioxidant defense, hypoxia activates p53 to suppress SLC7A11 expression, thereby reducing cystine uptake and GSH synthesis and consequently attenuating GPX4 antioxidant capacity; hypoxia also directly inhibits GPX4 activity, leaving PUFAs vulnerable to oxidation into PE-PUFA-OOH. Additionally, mitochondrial GPX4 cooperates with the coenzyme Q_10_ (CoQ_10_)/dihydroorotate dehydrogenase (DHODH) system to suppress ROS production, and hypoxia-mediated downregulation of this pathway further exacerbates oxidative stress. Within the Nrf2 signaling axis, hypoxia activates Nrf2 nuclear translocation to induce HO-1 expression, and HO-1-mediated Fe^2+^ release establishes a positive feedback loop. These converging pathways synergistically promote ferroptosis. Legend: → (Solid arrow): Activation/Promotion. Indicates that the upstream molecule promotes or activates the function of the downstream molecule. ├ (T-bar): Inhibition. Indicates that the upstream molecule inhibits or negatively regulates the downstream molecule.

Magnetic resonance imaging follow-up at 4 days and 5 months after percutaneous coronary intervention (PCI) in 48 ST-segment elevation myocardial infarction (STEMI) patients revealed persistent iron deposition in infarcted and left ventricular remodeling areas [93]. Animal studies showed that ferroptosis-related indicators (ACSL4, GPX4, iron, and malondialdehyde) remained stable during myocardial ischemia, but ACSL4, iron, and malondialdehyde levels increased with concurrent GPX4 downregulation after reperfusion, indicating that the reperfusion phase is a critical window for ferroptosis activation [94]. Mechanisms involving iron metabolism dysregulation include the following: TFR1 activation enhances ferritinophagy [95], promoting intracellular iron accumulation, whereas the NRF2/FPN1 signaling pathway suppresses ferroptosis by regulating iron homeostasis, and its activation alleviates MI/RI [96]. Interventional studies demonstrated that iron chelators, glutamine metabolism inhibitors, or specific ferroptosis inhibitors (e.g., lipopeptide inhibitor 1) can mitigate MIRI by modulating voltage-dependent anion channel 1 (VDAC1) and GPX4 expression [11,94]. Mechanistically, ALOX15 and its metabolite 15-HpETE promote PGC1-α ubiquitination and degradation (mediated by RNF34), impair mitochondrial biogenesis, and exacerbate lipid peroxidation and ferroptosis [97,98], whereas cardiac FTH deletion upregulates anti-ferroptotic proteins like HO-1, inhibiting ferroptosis via SLC7A11 induction [99]. Additionally, the USP7/p53/TFR1 pathway contributes to ferroptosis regulation in MI/RI: USP7 activates TFR1 expression through p53 deubiquitination, promoting ferroptosis, while TFR1 knockout suppresses ferroptosis independently of p53 [100]. These findings collectively highlight the central role of ferroptosis in MI/RI and the potential for multi-target interventions (Figure 4).

#### 2.2.4. Doxorubicin-Induced Cardiomyopathy

DOX is a potent anthracycline-class anticancer agent that remains widely used in the treatment of breast cancer, leukemia, and various solid tumors [101]. However, its clinical application is limited by dose-dependent cardiotoxicity, which can lead to irreversible degenerative cardiomyopathy and heart failure [102,103,104]. Notably, over 25% of patients receiving a cumulative dose of 550 mg/m^2^ doxorubicin develop heart failure [105]. Previous studies have confirmed that ferroptosis plays a critical role in the pathogenesis of doxorubicin-induced cardiotoxicity (DIC) [106].

**Figure 5:**
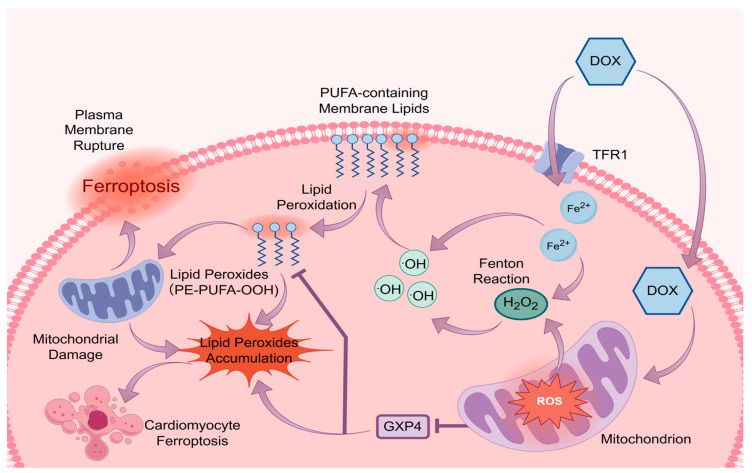
The characteristics of ferroptosis in Doxorubicin -Induced Cardiomyopathy. This figure presents the cellular mechanisms of ferroptosis in doxorubicin (DOX)-induced cardiomyopathy. DOX facilitates Fe^2+^ entry into cardiomyocytes via the TFR1 receptor; free Fe^2+^ subsequently catalyzes H_2_O_2_ decomposition through the Fenton reaction, generating abundant hydroxyl radicals (·OH) and ROS. Within mitochondria, DOX induces ROS burst while concurrently suppressing GPX4 antioxidant activity. ROS attack PUFA-containing membrane lipids, initiating a lipid peroxidation cascade that produces phospholipid hydroperoxides (PE-PUFA-OOH). The massive accumulation of lipid peroxides within mitochondria causes mitochondrial damage, which progresses to plasma membrane rupture and culminates in cardiomyocyte ferroptosis. Legend: → (Solid arrow): Activation/Promotion. Indicates that the upstream molecule promotes or activates the function of the downstream molecule. ├ (T-bar): Inhibition. Indicates that the upstream molecule inhibits or negatively regulates the downstream molecule.

Studies have demonstrated that DOX induces cardiotoxicity, accompanied by increased cardiac iron levels, accumulation of lipid-derived reactive oxygen species (ROS), and upregulation of ferroptosis biomarkers. Fang et al. revealed that ferroptosis contributes to DIC, and its inhibition exerts cardioprotective effects [10]. Liu et al. further identified acyl-CoA thioesterase 1 (ACOT1) as a ferroptosis suppressor, suggesting its regulatory role as a potential therapeutic strategy for DIC [107]. Additionally, ALOX15 modulates ferroptosis via the ROS-mediated MAPK signaling pathway during lipid peroxidation, indicating its potential as a therapeutic target for DIC intervention [108]. Tadokoro et al. found that DOX suppresses GPX4 and induces LPO, leading to mitochondrial-dependent ferroptosis, while the ferroptosis inhibitor ferrostatin-1 (Fer-1) alleviates DOX-induced cardiomyocyte injury [109]. Zhang et al. reported that DOX upregulates high mobility group box 1 (HMGB1) expression, promoting ferroptosis-related cardiotoxicity, which can be reversed by Fer-1 or dexrazoxane (DXZ) [110]. Other studies showed that FUNDC2 knockout mitigates DOX-induced cardiac dysfunction and myocardial fibrosis by suppressing mitochondrial morphological alterations and ferroptosis both in vivo and in vitro. Mechanistically, FUNDC2 interacts with the mitochondrial glutathione transporter SLC25A11, reducing mitochondrial GSH levels and triggering lipid peroxidation and ferroptosis [111]. Furthermore, DOX treatment significantly decreases antioxidant levels, including GPX4, superoxide dismutase (SOD), and GSH, exacerbating lipid peroxidation and ferroptosis [112,113]. Wu et al. identified glutathione S-transferase P1 (GSTP1) as a DOX target; its overexpression attenuates DIC by suppressing JNK phosphorylation, reducing ROS production, and inhibiting ACSL4-dependent ferroptosis [114]. In summary, targeting ferroptosis represents a promising therapeutic approach for DIC (Figure 5).

#### 2.2.5. Septic Cardiomyopathy

Sepsis is a life-threatening organ dysfunction caused by a dysregulated host response to infection. Approximately 70% of sepsis patients develop septic cardiomyopathy (SCM), which represents a major contributor to sepsis-related morbidity and mortality [115,116]. Previous studies have indicated that lipopolysaccharide (LPS) or stimulator of interferon genes (STING) activation directly participates in sepsis-induced cardiac dysfunction by inducing apoptotic autophagy, pyroptosis, or necroptosis. Inhibition of LPS- or STING-induced regulated cell death has been shown to partially attenuate sepsis-related myocardial injury [117,118,119,120].

**Figure 6:**
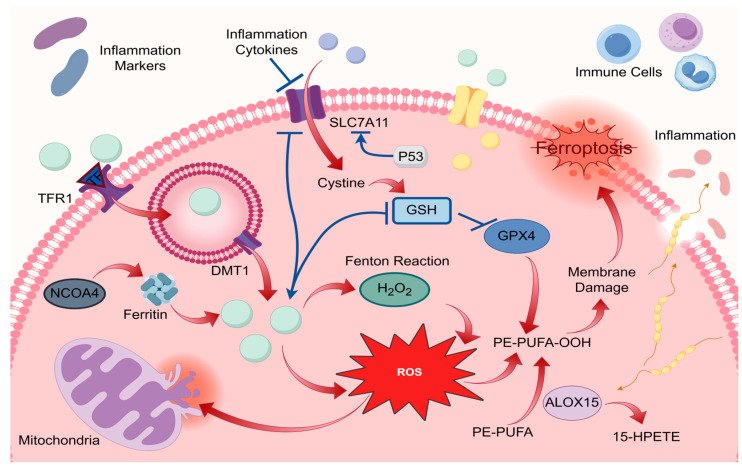
The characteristics of ferroptosis in Septic cardiomyopathy. This figure depicts the complex regulatory network interconnecting ferroptosis and inflammatory responses in septic cardiomyopathy. Inflammatory cytokines suppress SLC7A11-mediated cystine uptake, a process in which p53 also participates, leading to insufficient GSH synthesis and diminished GPX4 activity. In iron metabolism, TFR1-mediated iron endocytosis, DMT1-mediated iron transport, and NCOA4-mediated ferritinophagy collectively elevate intracellular free Fe^2+^ levels; Fe^2+^ then generates ROS via the Fenton reaction. In the lipid peroxidation pathway, ALOX15 catalyzes PE-PUFA oxidation to generate 15-hydroperoxyeicosatetraenoic acid (15-HPETE), which is further converted to PE-PUFA-OOH, while ROS directly attack the plasma membrane to induce membrane damage. Ferroptosis, once initiated, releases inflammatory mediators and recruits immune cells, thereby establishing a “ferroptosis-inflammation” vicious cycle that ultimately amplifies myocardial injury in septic cardiomyopathy. Legend: → (Solid arrow): Activation/Promotion. Indicates that the upstream molecule promotes or activates the function of the downstream molecule. ├ (T-bar): Inhibition. Indicates that the upstream molecule inhibits or negatively regulates the downstream molecule.

Ferroptosis has been identified as a key mechanism in the pathogenesis of SCM. Shen and Frazier reported elevated cyclooxygenase-2 (COX2, a recognized ferroptosis marker) in hearts of septic mice [121,122]. Li et al. further demonstrated that LPS upregulates NCOA4 expression, promoting ferritin degradation via NCOA4-mediated ferritinophagy, which expands the LIP and ultimately induces ferroptosis [123]. Notably, ferroptosis inhibitors deferoxamine and Fer-1 reduced ferroptosis in cardiomyocytes, improved cardiac function, and decreased mortality in septic mice. In a cecal ligation and puncture (CLP)-induced sepsis model, Wang et al. observed increased cardiac troponin I levels alongside significant reductions in GPX4 and GSH [124]. Transmembrane protein 43 (TMEM43), a cardiomyopathy-associated protein, exerts cardioprotective effects by suppressing LPS-induced LPO: TMEM43 overexpression inhibits ferroptosis and alleviates cardiac injury, whereas its knockdown exacerbates LPS-induced cardiomyopathy, accompanied by enhanced ferroptosis, downregulated p53 and ferritin, and dysregulated GPX4/SLC7A11 expression. Fer-1 treatment ameliorated the aggravated cardiac injury induced by TMEM43 knockdown [125,126]. Collectively, these findings indicate that ferroptosis is a critical pathogenic factor in SCM, and targeting this pathway may represent a promising therapeutic strategy (Figure 6).

In summary, ferroptosis profoundly contributes to the pathological progression of cardiovascular diseases through multidimensional molecular mechanisms, including dysregulated iron metabolism, uncontrolled lipid peroxidation, and suppression of the antioxidant system. Its aberrant activation serves as a critical driver of cardiomyocyte death, inflammatory responses, and ventricular remodeling. Delineating the regulatory network of ferroptosis in cardiac pathologies will provide a theoretical foundation for developing targeted therapeutic strategies.

## 3. Material Basis and Molecular Mechanisms of Traditional Chinese Medicine in Regulating Ferroptosis

TCM exhibits unique advantages in regulating ferroptosis due to its synergistic therapeutic characteristics of “multi-component, multi-target, and multi-pathway” modulation. Its material basis encompasses active ingredients from single herbs (such as flavonoids, polyphenols, and alkaloids), complex component networks of compound formulations, as well as the physical stimulation effects of characteristic therapies like acupuncture. The mechanisms of action involve multi-dimensional regulation of iron metabolism, the antioxidant system (e.g., GPX4), key lipid-metabolizing enzymes (e.g., ACSL4), and nuclear transcription factors (e.g., Nrf2, p53), collectively forming a multi-level and systematic anti-ferroptosis network.

### 3.1. Active Ingredients of Single Medicinal Herbs: Key “Tools” for Targeted Regulation of Core Ferroptosis Pathways

The active compounds derived from single medicinal herbs serve as the direct material basis for regulating ferroptosis. They function by precisely targeting key molecules within the core ferroptosis pathways (such as GPX4, System Xc^−^, and ACSL4), thereby restoring the balance between “pro-death” and “anti-death” mechanisms.

#### 3.1.1. Flavonoids Compounds

Flavonoids play significant roles in cardiovascular protection. Baicalin modulates the SENP1/SIRT3 signaling pathway, inhibits SIRT3 SUMOylation, restores mitochondrial dynamics and autophagy, thereby ameliorating ferroptosis and apoptosis in DbCM [127]. Wogonin markedly alleviates cardiac inflammation, oxidative stress, and mitochondrial dysfunction in SCM models, primarily by suppressing ALOX15-mediated lipid peroxidation and ferroptosis [128]. Naringenin (NRG) activates the PI3K/AKT pathway and the Nrf2/system xc^−^/GPX4 axis, mitigating oxidative stress, autophagy, and ferroptosis in MI/RI [129,130]. In addition, Jian et al. indicated that NRG was able to relieve DOX-induced cardiotoxicity by suppressing myocardial apoptosis via the p38MAPK pathway [131]. Isoliquiritigenin (ISL) reduces intracellular labile iron accumulation via the Nrf2/HO-1 pathway, upregulates GPX4 and SLC7A11 expression, and inhibits lipid peroxidation, thus attenuating ferroptosis-related myocardial injury [132,133]. Licochalcone A (Lico A) suppresses p53 activity through the PI3K/AKT/MDM2 axis, promotes SLC7A11 and GPX4 expression, and counteracts DOX -induced cardiac ferroptosis [134]. Kaempferol (KP) and Galangin (Gal) inhibit DOX-triggered mitochondrial ROS-dependent ferroptosis via the Nrf2/SLC7A11/GPX4 and GSTP1/JNK pathways, respectively [135,136]. Total flavonoids from Clinopodium chinense (TFCC) and tournefolic acid B (TAB) exert anti-ferroptotic and cardioprotective effects by activating the PI3K/AKT/Nrf2/HO-1 pathway or suppressing ERS [137,138,139]. Collectively, these findings highlight the multi-target regulatory capacity of flavonoids against ferroptosis, offering promising therapeutic strategies for cardiovascular diseases (Table 1).

#### 3.1.2. Glycosides Compounds

Glycosides exhibit multi-pathway mechanisms in cardiovascular protection. Ginsenoside Rg3 activates the keap1/Nrf2/GPX4 signaling pathway to suppress ferroptosis in MI/RI, reduces iron deposition, and improves cardiac function [140,141]; its isomer 20(R)-ginsenoside Rg3 also alleviates mitochondrial oxidative stress via the Nrf2/HO-1 pathway [142]. Ginsenoside Rg1 inhibits autophagy and ERS, ameliorating DOX-induced cardiotoxicity and apoptosis in DbCM [143,144]. Ginsenoside Rb1 attenuates acute DIC-related myocardial injury by regulating autophagy and ferroptosis through the AMPK and Nrf2 signaling pathways, respectively [145,146]. Diosgenin exerts cardioprotective effects through multiple mechanisms: it suppresses oxidative damage via the miR-140-5p/Nrf2/Sirt2 axis [147], inhibits apoptosis and excessive autophagy through the PDKa1/AKT/mTOR pathway [148], and counteracts DOX-induced ferroptosis via the Nrf2/GPX4 axis and downstream iron metabolism genes [149]. Additionally, it blocks ER stress-mediated ferroptosis by suppressing markers such as PERK/eIF2α phosphorylation [150]. Salidroside reduces mitochondrial dysfunction and lipid peroxidation in an AMPK-dependent manner to inhibit ferroptosis [151]. Astragaloside IV (AS-IV) alleviates myocardial injury in DbCM by inhibiting CD36-mediated lipid accumulation and ferroptosis [152]. In addition, it can also inhibit adramycin-induced cardiac iron necrosis by enhancing Nrf2 signaling [153]. Pedunculoside activates the Nrf2/HO-1 pathway, mitigating high glucose-induced oxidative stress, inflammation, and apoptosis [154]. Sarmentosin, a rare cyanogenic glycoside alkaloid, alleviates ferroptosis-related myocardial injury in DOX-induced cardiotoxicity models by activating autophagy and the Nrf2 signaling pathway, significantly suppressing iron deposition, lipid peroxidation, and oxidative stress [155]. Otherwise, pretreatment with Panaxatriol Saponin attenuates mitochondrial apoptosis and oxidative stress to facilitate treatment of MI/RI via the regulation of Keap1/Nrf2 Activity [156]. Collectively, these findings demonstrate that glycosides confer synergistic cardioprotection by modulating ferroptosis, oxidative stress, autophagy, and ER stress pathways (Table 2).

#### 3.1.3. Phenolic Compounds

Phenolic compounds exert cardiovascular protection through multi-pathway regulatory mechanisms. Yellow wine polyphenol compounds (YWPC) activate the Nrf2 pathway to suppress oxidative stress, ameliorating DOX-induced mitochondrial damage, fibrosis, and cardiac dysfunction [157]. Astragalus polyphenols enhance mitochondrial membrane potential, reduce ROS levels, and downregulate ANP/BNP expression via the PI3K/AKT/Nrf2 axis, alleviating DIC-related myocardial injury [158]. Resveratrol (Res) inhibits ferroptosis by modulating the p62-Nrf2 axis and MAPK signaling, with efficacy comparable to Fer-1, and attenuates MI/RI through the AMPK/p38/Nrf2 pathway [159,160,161]. Caffeic acid phenethyl ester (CAPE) suppresses oxidative stress and necroptosis via ROS-MLKL signaling crosstalk [162]. 6-Gingerol (6-G) enhances SOD activity, reduces MDA levels, and suppresses FACL4 expression and iron accumulation via the Nrf2/HO-1 pathway, thereby improving ferroptosis and inflammation in DbCM [163]. Punicalagin (PUN) counteracts DOX-induced oxidative stress by promoting Nrf2 nuclear translocation and HO-1 expression [164]. Curcumin activates the Nrf2/GPX4/HO-1 axis to inhibit glucose- or erastin-triggered ferroptosis and delays DCM progression through the AKT/Nrf2/ARE pathway [73,165,166]. Collectively, these findings highlight that phenolic compounds primarily modulate the Nrf2 pathway and its downstream antioxidant effects, synergistically inhibiting ferroptosis, oxidative stress, and apoptosis, offering multi-target strategies for cardiovascular diseases (Table 3).

#### 3.1.4. Polysaccharide Compounds

Polysaccharides demonstrate significant ferroptosis-regulating potential in cardiovascular protection. Fucoidan, a sulfated water-soluble polysaccharide [167], alleviates DOX-induced cardiotoxicity by promoting nuclear translocation of Nrf2, upregulating transcriptional expression of GPX4, and subsequently suppressing ROS accumulation and lipid peroxidation while restoring iron homeostasis [168]. Ophiopogon japonicus polysaccharide (OJP) exerts concentration-dependent anti-ferroptotic effects by enhancing Nrf2 expression, activating downstream GPX4, reducing TFR1 levels, decreasing labile iron pool accumulation and lipid peroxidation products, and improving mitochondrial function [169,170]. Both polysaccharides primarily act through the Nrf2/GPX4 signaling axis, highlighting their potential as natural therapeutic agents against chemotherapy-associated cardiac injury. Additionally, in a DOX-induced cardiotoxicity model, Astragalus polysaccharides were shown to restore autophagic flux in cardiomyocytes by modulating the AMPK/mTOR signaling pathway, thereby attenuating excessive autophagy-mediated cell death and ultimately exerting cardioprotective effects [171]. Lycium barbarum polysaccharide (LBP) exhibited a cardiac protective effect on the ischemic myocardium of rats after reperfusion and attenuated myocardial I/R injury via autophagy inhibition-induced Nrf2 activation [172] (Table 4).

#### 3.1.5. Terpenoid Compounds

Terpenoids exert cardioprotective effects through multi-pathway regulatory mechanisms. Salvia miltiorrhiza (Danshen) activates the Nrf2 signaling pathway, upregulating xCT and GPX4 expression, reducing ferrous iron and malondialdehyde (MDA) levels, while enhancing GSH content and SOD activity, thereby suppressing ferroptosis after myocardial infarction with efficacy comparable to Fer-1 [173]. Tanshinone I (Tan I), as an Nrf2 agonist, mitigates oxidative stress and preserves mitochondrial function via this pathway, alleviating DOX-induced cardiotoxicity [174]. Tanshinone IIA inhibits histone deacetylase 1 (HDAC1) expression, activating the Nrf2-xCT/GPX4/HO-1 axis to reduce infarct size, apoptosis, inflammation, and ferroptosis in MI/RI models [175]. Dihydroartemisinin (DHA) not only activates Nrf2 but also enhances autophagic flux and lysosomal function, synergistically inhibiting DOX-triggered oxidative stress and ferroptosis [176]. Additionally, Artemisinin alleviates arsenic-induced myocardial injury by maintaining extracellular matrix homeostasis and modulating oxidative stress/inflammatory responses [177]. These findings indicate that terpenoids primarily act through the Nrf2 pathway, coupled with autophagy regulation and oxidative stress suppression, providing multi-dimensional cardioprotection (Table 5).

#### 3.1.6. Lignan Compounds

Lignans modulate ferroptosis through specific molecular mechanisms. Schisandrol B regulates redox homeostasis and energy metabolism via the p53/SLC7A11/GPX4 signaling axis in DbCM models, preserving mitochondrial integrity and function while reducing lactate dehydrogenase (LDH) release and suppressing ferroptosis [178]. Protosappanin A exerts cardioprotection through a dual mechanism: directly binding to ACSL4 and FTH1 to inhibit ACSL4 phosphorylation and block FTH1 autophagic degradation, while simultaneously modulating immune responses via NF-κB and AKT/mTOR pathways, collectively alleviating DOX-induced mitochondrial dysfunction and ferroptosis-related myocardial injury [179]. Additionally, Honokiol (HKL) enhances antioxidant capacity via the SIRT1-Nrf2 signaling cascade, reducing cardiomyocyte apoptosis and infarct size [180] (Table 6).

#### 3.1.7. Aldehyde Compounds

Cinnamaldehyde (CA), an α,β-unsaturated aromatic aldehyde, demonstrates significant cardioprotective activity. Studies show that CA alleviates DOX-induced myocardial injury by regulating key oxidative stress indicators: in DOX-treated H9c2 cells and rat cardiomyocyte models, CA effectively reduces ROS and MDA levels while enhancing SOD activity and GSH content. Mechanistically, CA promotes nuclear translocation of Nrf2, leading to upregulated HO-1 expression and subsequent suppression of cardiomyocyte ferroptosis. Notably, the ferroptosis inducer Erastin reverses CA-mediated protection of cardiomyocyte viability, confirming the essential role of Nrf2/HO-1 pathway activation in CA’s anti-ferroptotic effects [181] (Table 7).

#### 3.1.8. Other Species

Beyond classical natural products, other structurally distinct compounds demonstrate significant cardioprotective potential. The primary active components of Prunella vulgaris (PV) mitigate MI/R by inhibiting oxidative stress and ferroptosis via the Nrf2/GPX4 pathway, with ursolic acid identified as the key constituent mediating these antioxidant and anti-ferroptotic effects [182]. These findings highlight the therapeutic potential of structurally diverse natural products targeting ferroptosis pathways in cardiac injury.

### 3.2. Herbal Formulations and Compound Preparations: A “Network Regulation” Model of Multi-Component Synergy

TCM formulations achieve a complementary composition and synergistic targeting network through the combination of multiple medicinal herbs. Their anti-ferroptosis effects are often more potent than those of single herbs and better aligned with the complex pathological demands of clinical practice.

#### 3.2.1. Guanxinning Injection

Guanxinning injection (GXN) is a Chinese clinical prescription refined from the aqueous extract of *Salvia miltiorrhiza* Bge. (Danshen) and *Ligusticum chuanxiong* Hort. (Chuanxiong) at a ratio of 1:1 (*w*/*w*). It has been used in China for almost twenty years to treat angina, heart failure (HF), and chronic kidney disease. This study investigated the role of GXN on renal dysfunction and fibrosis in heart failure mice and its regulation of the SLC7A11/GPX4 axis. The transverse aortic constriction (TAC) model was used to induce HF accompanied by kidney fibrosis. GXN was administered via tail vein injection at doses of 12.0, 6.0, and 3.0 mL/kg. The results demonstrated that GXN significantly maintained cardiac function (ejection fraction, cardiac output, left ventricle volume) and alleviated the progression of renal fibrosis (serum creatinine, collagen volume fraction, connective tissue growth factor) in heart failure mice. Metabolomic analysis revealed that GXN regulated redox metabolism pathways, including aspartate, glycine, serine, and cystine metabolism. Furthermore, GXN was found to increase catalase content and upregulate the expression of GPX4, SLC7A11, and FTH1 in the kidney, while downregulating xanthine oxidase and nitric oxide synthase contents. The study identified 35 chemical constituents in GXN, and network pharmacology analysis suggested that the cardio-renal protective effect may be attributed to multi-components such as rosmarinic acid, caffeic acid, ferulic acid, senkyunolide E, protocatechualdehyde, protocatechuic acid, danshensu, L-Ile, vanillic acid, and salvianolic acid A [183].

#### 3.2.2. YiQi FuMai Injection

The YiQi FuMai injection (YQFM) is a traditional Chinese medicine composed of *Panax ginseng* C.A. Mey. (Renshen), *Ophiopogon japonicus* (L. f.) Ker-Gawl. (Maidong), and *Schisandra chinensis* (Turcz.) Baill. (Wuweizi), widely used in China to treat cardiovascular diseases such as coronary heart disease, heart failure, and SCM. It has effects on alleviating myocardial injury, reducing oxidative stress, and improving cardiac function. Guo et al. investigated the regulatory mechanism of YQFM on ferroptosis in SCM rats and LPS-induced H9c2 cells. The results demonstrated that YQFM improved cardiac function in septic model rats by reducing iron overload and lipid peroxidation (ROS, MDA, 4-HNE), upregulating the xCT/GPX4 axis (including SLC7A11, SLC3A2, and GPX4), and subsequently inhibiting ferroptosis both in vivo and in vitro [184].

#### 3.2.3. Qishen Granule

The TCM Compound Qishen Granule (QSG) is composed of six herbs: *Astragalus membranaceus* (Fisch.) Bge. (Huangqi), *Salvia miltiorrhiza* Bge. (Danshen), *Lonicera japonica* Thunb. (Jinyinhua), *Aconitum carmichaelii* Debx. (fuzi), *Scrophularia ningpoensis* Hemsl. (Xuanshen) and *Glycyrrhiza uralensis* Fisch. (Gancao). Developed from the traditional formula “Zhenwu Tang (ZWT)”, it is widely used in TCM for treating heart diseases and has been frequently reported to improve cardiac function and reduce fibrosis [185,186,187]. Clinical studies have confirmed that QSG is significantly effective and safe in treating chronic heart failure [188,189]. Preliminary research indicates that QSG alleviates DOX-induced mitochondrial oxidative damage and apoptosis by coordinating mitophagy and mitochondrial biogenesis [190]. Another study found that QSG ameliorates oxidative damage and partially preserves mitochondrial function by restoring protein acetylation levels and activating the SIRT3/Ac-SOD2 pathway. Furthermore, Xue et al. investigated the anti-ferroptotic effects of QSG through in vivo and in vitro experiments and explored whether QSG could alleviate DIC by acting on the Nrf2 pathway. In vivo, QSG improved cardiac function in DIC mice, inhibited DOX-induced accumulation of labile iron and MDA, increased GSH levels post-intervention, and prevented DOX-induced mitochondrial structural damage. Concurrently, it promoted the expression of proteins associated with the Nrf2 pathway, thereby counteracting ferroptosis. In vitro, QSG facilitated the nuclear translocation of Nrf2. Interference with Nrf2 attenuated QSG’s regulatory effects on labile iron content and mitochondrial reactive oxygen species (ROS) production. Additionally, Nrf2 knockdown weakened the anti-ferroptotic effect of QSG and suppressed the expression of key proteins in the Nrf2 pathway [191]. These results demonstrate that QSG alleviates DIC by inhibiting ferroptosis via the Nrf2 pathway.

#### 3.2.4. Zhilong Huoxue Tongyu Capsule

Zhilong Huoxue Tongyu Capsule (ZL) is a TCM formulation used for treating cardiovascular and cerebrovascular diseases. It is composed of five herbs in a specific ratio: *Astragalus membranaceus* (Fisch.) Bge. (Huangqi, 8 parts), *Pheretima aspergillum* (E. Perrier) (Dilong, 4 parts), *Sargentodoxa cuneata* (Oliv.) Rehd. et Wils. (Daxueteng, 4 parts), *Cinnamomum cassia* Presl (Guizhi, 3 parts), and *Whitmania pigra* Whitman (Shuizhi, 1 part) [192]. Previous studies have shown that administration at the maximum tolerated dose in mice (81.6 g/kg/d) did not induce significant adverse effects on physiological functions or long-term toxicity [193]. Furthermore, ZL has been demonstrated to effectively attenuate cerebral ischemia–reperfusion injury [192]. Building on this foundation, Zhao et al. further investigated the impact of ZL on MI/RI-induced ferroptosis via regulation of the PI3K/AKT/Nrf2 signaling pathway, aiming to elucidate the mechanism underlying its cardioprotective effects. The results revealed that ZL prevents MI/RI-induced ferroptosis by modulating the PI3K/AKT signaling pathway, leading to increased Nrf2 expression and subsequent activation of the HO-1/GPX4 pathway [194]. These findings illuminate the potential therapeutic mechanism of ZL in cardiovascular diseases.

#### 3.2.5. QiShenYiQi Dripping Pill

QiShenYiQi dripping pill (QSYQ) is a Chinese herbal formula composed of *Astragalus membranaceus* Fisch. ex Bunge (Huangqi), *Salvia miltiorrhiza* Bunge (Danshen), *Panax notoginseng* (Burkill) F.H. Chen (Sanqi), and *Dalbergia odorifera* T.C. Chen (Jiangxiang) in a proportion of 10:5:1:0.067. It was approved by the National Medical Products Administration (NMPA) in 2003 and is used clinically for ischemic heart diseases. Wu et al. investigated the regulatory mechanism of QSYQ on myocardial ischemia-induced ferroptosis in mice. The results demonstrated that QSYQ alleviated myocardial ischemia injury by improving cardiac function, reducing lipid peroxidation and iron content, and restoring mitochondrial ultrastructure. Mechanistically, QSYQ promoted mitochondrial biogenesis (via upregulating PGC-1α, Nrf1, and TFAM) and mitochondrial dynamic homeostasis (by enhancing fusion proteins MFN-2 and OPA1 while inhibiting excessive fission through reduced phosphorylation of Drp1 at Ser616), thereby inhibiting ferroptosis in cardiomyocytes both in vivo and in vitro [195].

#### 3.2.6. Ling-Gui-Zhu-Gan Decoction

Ling-Gui-Zhu-Gan decoction (LGZGD) is a classic formula from Zhang Zhongjing’s Han Dynasty medical text, composed of four herbs: *Poria cocos* (Schw.) Wolf (Fuling), *Cinnamomum cassia* Presl (Guizhi), *Atractylodes macrocephala* Koidz. (Baizhu), and *Glycyrrhiza uralensis* Fisch. (Gancao). It has been widely used to improve cardiac function and alleviate myocardial injury. Yang et al. investigated the protective mechanism of LGZGD against doxorubicin (DOX)-induced myocardial injury in rats and H9c2 cells [196]. The results demonstrated that LGZGD improved cardiac systolic function, reduced oxidative stress markers (MDA, LDH, CK), increased antioxidant enzyme SOD activity, and upregulated Nrf2, GPX4, and Fpn1 expression while downregulating Ptgs2. Further experiments showed that LGZGD inhibited ferroptosis in cardiomyocytes by activating the Nrf2 signaling pathway, thereby ameliorating DOX-induced cardiotoxicity.

#### 3.2.7. HJ11 Decoction

HJ11 decoction is a novel traditional Chinese medicine developed from the appropriate addition and reduction of Si-Miao-Yong-An decoction; it consists of components such as *Lonicera japonica* Thunb. (Jinyinhua) and *Scrophularia ningpoensis* Hemsl. (Xuanshen), among others (with specific ingredients currently in the confidential stage). It has been commonly used to treat I/R injury in the clinical setting and has shown protective effects against myocardial I/R injury. Zhang et al. investigated the regulatory mechanism of HJ11 decoction on myocardial I/R injury in rats [197]. The results demonstrated that HJ11 decoction improved cardiac function; attenuated myocardial injury, apoptosis, oxidative stress, mitochondrial damage, and iron accumulation; and reduced infarct size in the myocardial I/R injury rat model. Mechanistically, HJ11 decoction suppressed the expression of ferroptosis-promoting proteins (ACSL4 and COX2) and promoted the expression of ferroptosis-inhibiting proteins (FTH1 and GPX4), thereby restraining the development of myocardial I/R injury by suppressing ACSL4-mediated ferroptosis (Table 8).

### 3.3. Characteristic TCM Therapies: Systemic Regulation Mediated by Physical Stimulation

In addition to herbal medicine, characteristic TCM therapies such as acupuncture and moxibustion exert regulatory effects through physical stimulation of acupoints, modulating the neuro-endocrine-immune network and indirectly influencing ferroptosis-related signaling pathways, demonstrating unique interventional advantages.

Electroacupuncture (EA), originating from TCM, has emerged as a non-pharmacological intervention involving electrical stimulation of specific acupoints. It has been shown to effectively alleviate symptoms and improve outcomes in various cardiovascular diseases, including chronic angina, heart failure, arrhythmias, DbCM, and MI/RI [198,199,200]. Jiang et al. [201] applied EA to the bilateral “Shenmen” (HT7) and “Tongli” (HT5) acupoints in a rat model of myocardial ischemic injury. They observed that EA treatment upregulated the expression of GSH, GPX4, and FTH1 in myocardial tissue and ameliorated myocardial cell alignment and fiber fragmentation. These findings demonstrate that EA can inhibit cardiomyocyte ferroptosis and improve myocardial ischemic injury, thereby effectively preventing the onset and progression of chronic heart failure (CHF). Similarly, Xiao et al. [202] experimentally verified that EA preconditioning exerts a protective effect against MI/RI in rats. The underlying mechanism is likely associated with the suppression of ferroptosis via the mTOR/ROS signaling pathway. This process significantly elevated the expression of GPX4 and FTH1, inhibited ferroptosis, and reduced myocardial damage. Furthermore, EA notably improved cardiac function by increasing the left ventricular ejection fraction (LVEF) and fractional shortening (FS), thereby preventing the development of CHF. Furthermore, Li et al. investigated the effect of EA on ferroptosis in a murine MI/RI model. Compared to the MI/RI group, the EA-treated group showed significantly improved cardiac function, reduced cardiac iron deposition, and elevated Nrf2 and HO-1 levels, indicating that EA alleviates ferroptosis-induced MI/RI via the Nrf2/HO-1 pathway [203]. Additionally, EA preconditioning effectively ameliorates MI/RI-induced myocardial injury through Nrf2-mediated suppression of oxidative stress and pyroptosis [204]. Another study by Liu et al. [205] demonstrated that EA at “Neiguan” (PC6) with varying intensities (0.5 mA, 1 mA, and 2 mA) during the reperfusion phase effectively ameliorated MI/RI in rats. The underlying mechanism appears to involve the activation of the Nrf2-GPX4 signaling axis, which inhibits ferroptosis—evidenced by decreased levels of Fe^2+^ and ACSL4, alongside increased expression of Nrf2, GPX4, and FTH1. Concurrently, EA attenuated oxidative stress, as indicated by reduced ROS and MDA levels and elevated SOD activity and GSH levels, and mitigated inflammatory responses by downregulating TNF-α, IL-6, and IL-1β while upregulating IL-10, thereby conferring cardioprotection. Furthermore, another experiment [203] demonstrated that EA exerts a protective effect against MI/RI. This effect is achieved by activating the Nrf2/HO-1 signaling pathway, which upregulates the expression of Nrf2 and HO-1, reduces myocardial iron deposition and MDA levels and decreases intracellular ROS. Concurrently, EA increases the expression of GPX4, thereby inhibiting cardiomyocyte ferroptosis induced by MI/RI and ultimately improving cardiac function.

In another study, Yan et al. examined the effect of moxibustion on antioxidant stress in DOX-treated rats. Moxibustion significantly improved left ventricular function and heart rate in cardiomyopathic rats, likely attributable to reduced myocardial oxidative stress via decreased MDA content and increased cardiac SOD activity [206]. Moxibustion also enhanced cardiac function in CHF rats by upregulating cardiomyocyte autophagy-related proteins [207,208]. Gao et al. [209] discovered that moxibustion applied to the “Xinshu” (BL15) and “Feishu” (BL13) acupoints could suppress atrial fibrosis in CHF rats by modulating autophagy to inhibit ferroptosis. Mechanistically, this intervention downregulated the expression of TfR1 and upregulated FSP1, leading to a reduction in total myocardial iron content. Consequently, the expression of atrial natriuretic peptide (ANP) and collagen type I (Col I) was suppressed, which slowed the progression of myocardial fibrosis and improved cardiac function. Additionally, Zhang et al. [210] experimentally demonstrated that moxibustion protects against cerebral ischemia–reperfusion injury (CI/RI) in rats. The underlying mechanism involves inhibiting ferroptosis by reducing lipid peroxide production, promoting the expression of GSH and GPX4, and suppressing the accumulation of ROS.

In summary, TCM exerts anti-ferroptotic effects through multiple dimensions—iron metabolism, lipid metabolism, and the antioxidant system—via targeted active components of single herbs, synergistic multi-component networks of compound formulations, and systemic regulation by characteristic therapies. These provide a substantial material basis and molecular mechanistic foundation for preventing and treating cardiovascular diseases. In-depth analysis of these “component-target-pathway” networks will offer critical theoretical support for developing efficient and low-toxicity TCM-based anti-ferroptosis drugs.

## 4. Challenges and Future Research Directions

A significant proportion of the studies included in this review utilized the H9C2 rat cardiomyoblast cell line as the primary in vitro experimental platform. As one of the most widely adopted models in cardiovascular research, H9C2 cells offer distinct advantages, including robust proliferative capacity, well-established culture protocols, high experimental reproducibility, and cost-effectiveness. These attributes render them an efficient tool for high-throughput screening and preliminary mechanistic exploration, particularly for validating biological processes such as ferroptosis. While the aforementioned studies provide robust evidence from cell culture models (e.g., H9c2 and AC16 cells) demonstrating that monomers like Baicalin and Wogonin can effectively inhibit ferroptosis via Nrf2/xCT pathways, these findings are primarily mechanistic. In vivo validation in animal models, such as the DOX-induced cardiotoxicity mice treated with Qishen Granule or the MI/RI rats treated with Zhilong Huoxue Tongyu Capsule, offers stronger preclinical support. These studies confirm that multi-herb formulations can ameliorate cardiac function and remodel the myocardial microenvironment by coordinately regulating iron metabolism and lipid peroxidation. However, it is critical to note that the current evidence is largely confined to preclinical stages. Although some formulas like Guanxinning Injection have clinical usage records, there is a paucity of high-quality randomized controlled trials (RCTs) specifically linking the anti-ferroptotic effects of TCM to clinical outcomes in cardiovascular diseases. Future investigations must bridge this gap by translating these promising animal data into rigorous clinical validations.

Beyond the intrinsic limitations of cellular models, a pervasive weakness in the primary literature is the inadequate reporting of compound concentrations and doses. Many studies describe TCM interventions using crude extract weights or “equivalent crude drug” quantities without specifying the concentration of active constituents, thereby precluding meaningful dose–response analyses or cross-study comparisons [211,212]. For monomeric compounds, the concentrations employed in vitro are rarely correlated with plasma levels achievable in vivo—a disconnect that is particularly severe for Curcumin and Resveratrol. The in vitro effective concentration range for Curcumin (10–50 μM) exceeds the in vivo free plasma Cmax by three to four orders of magnitude; Resveratrol faces a similar dilemma wherein experimental concentrations far surpass physiologically relevant exposure levels. This profound exposure–response disconnect decouples mechanistic insights from therapeutic reality.

More critically, Curcumin and Resveratrol—two of the most extensively studied TCM monomers—belong to the pharmacological class of Pan-Assay Interference Compounds (PAINS). These chemical entities produce false-positive readouts on diverse bioassays through nonspecific mechanisms, including colloidal aggregation, metal chelation, redox cycling, and membrane perturbation [213,214]. Curcumin exemplifies multiple PAINS structural alerts: its β-diketone moiety confers potent iron-chelating capacity; its catechol-like groups undergo autoxidation to generate reactive quinone intermediates; and its α,β-unsaturated carbonyl acts as a Michael acceptor capable of covalently modifying protein cysteine residues. In ferroptosis research, the iron-chelating property of Curcumin is especially concerning, as it may directly inhibit ferroptosis by chemically sequestering metal ions required for Fenton reactions—an effect distinct from the pharmacological modulation of ferroptosis regulatory pathways. At concentrations exceeding 10–20 μM, Curcumin forms colloidal aggregates in aqueous solution that nonspecifically adsorb and denature proteins. Resveratrol presents analogous issues: its planar, lipophilic structure enables membrane insertion and nonspecific modulation of membrane protein function, while its resorcinol moiety undergoes autoxidation to yield reactive intermediates [215].

This superposition of inadequate dose reporting and PAINS attributes creates a severe hermeneutic dilemma—investigators cannot ascertain whether in vitro concentrations fall within pharmacologically relevant exposure ranges, nor can they exclude the possibility that observed effects stem from nonspecific assay interference rather than specific pathway modulation. A significant proportion of the anti-ferroptotic effects attributed to these compounds may thus reflect PAINS-driven artifacts rather than specific pathway regulation, necessitating orthogonal validation via gene knockout, structure–activity relationship (SAR) analysis, and deaggregation controls to exclude confounding colloidal aggregation effects.

Despite the elucidation of TCM’s multi-tiered therapeutic arsenal against myocardial ferroptosis spanning active phytochemicals, classical formulae, and physical therapies, profound translational and methodological challenges critically undermine the field’s progress. Key limitations include mechanistic homogeneity and redundancy, where >90% of compounds converge on the Nrf2/HO-1/GPX4 axis without specificity; methodological flaws rooted in over-reliance on H9c2 embryonic cells and the absence of gold-standard Ferrostatin-1 rescue assays; biomarker ambiguity with non-specific indicators (ROS, MDA, Caspase-3) frequently confounded with specific drivers (PTGS2, 4-HNE); and significant translational barriers comprising PAINS (Pan-assay interference) artifacts (e.g., Curcumin’s ~1% bioavailability), herb-to-herb variability, and a glaring absence of head-to-head comparisons with clinically advanced synthetic Nrf2 modulators like Bardoxolone methyl.

Future research must therefore pivot toward a rigorously evidence-based and comparative paradigm: First, mechanistic validation must mandate Fer-1/Liproxstatin-1 rescue experiments and PAINS filtering to eliminate chemical artifacts, shifting focus from descriptive marker lists to functional GPX4 activity and lipidomics. Second, translational models should abandon H9c2 cells in favor of human iPSC-derived cardiomyocytes (hiPSC-CMs) and organoids, integrating single-cell/spatial transcriptomics to resolve spatiotemporal heterogeneity. Third, clinical translation requires establishing physiologically based PK/PD models for accurate Human Equivalent Dose (HED) conversion, utilizing nanodelivery systems (QbD principles) to overcome bioavailability issues, and conducting adaptive clinical trials that incorporate dynamic monitoring of ferroptosis-specific biomarkers (PTGS2, 4-HNE) to enable direct benchmarking against standard-of-care Nrf2 therapies. By embracing this multi-pronged strategy, TCM can transition from empirical observation to a precision medicine framework, providing safer and mechanistically validated strategies for ferroptosis-driven cardiovascular diseases.

## 5. Conclusions

Ferroptosis, an iron-dependent form of regulated cell death driven by lipid peroxidation, has been established as a core pathological link in the development and progression of various cardiovascular diseases, including ICM, DIC, DCM, DbCM and SCM. Its molecular regulatory network is intricate and sophisticated, primarily revolving around several central axes: the functional integrity of the GPX4/System Xc^−^/GSH antioxidant axis, the balance of iron metabolism homeostasis (involving TFR1, NCOA4-mediated ferritinophagy, and FPN1-mediated iron export), and the intensity of ACSL4/LPCAT3/ALOX15-mediated lipid peroxidation signaling. The aberrant activation of these pathways collectively leads to oxidative membrane damage and functional loss in cardiomyocytes.

This review systematically summarizes the latest research progress on the regulation of ferroptosis by TCM in cardiovascular diseases, highlighting its unique “systems biology” interventional advantage. Evidence indicates that TCM provides a multi-tiered therapeutic arsenal. Firstly, at the level of active components—ranging from flavonoids (e.g., Baicalin, Naringenin), glycosides (e.g., Ginsenoside Rg3, Astragaloside IV), and phenols (e.g., Resveratrol, Curcumin) to polysaccharides and terpenoids—although structurally diverse, these components converge on key nodes such as Nrf2, GPX4, and ACSL4. Through multi-target synergistic actions, they restore cellular redox homeostasis and iron metabolic balance. Secondly, at the compound formula level (e.g., Qishen Granule, Zhilong Huoxue Tongyu Capsule), the compatibility principle of “sovereign, minister, assistant, and envoy” manifests molecularly as a more comprehensive and synergistic regulation of the ferroptosis signaling network. The effects are often superior to those of single components and better aligned with the complex clinical pathophysiology. Furthermore, physical therapies like electroacupuncture inhibit ferroptosis by activating pathways such as Nrf2/HO-1, providing non-pharmacological intervention methods and further expanding the therapeutic dimensions of TCM.

However, this field still faces several profound challenges and unanswered scientific questions: First, the complexity of mechanistic crosstalk. Ferroptosis does not occur in isolation; it engages in complex interplay with other cell death modalities such as apoptosis, necroptosis, and autophagy. How TCM components precisely orchestrate the anti-ferroptosis program within this intertwined cell death signaling network—rather than merely inhibiting it—and the hierarchy and spatiotemporal specificity of these actions remain unclear. Second, drug delivery and targeting. Many active components suffer from issues like low oral bioavailability and widespread tissue distribution. Developing strategies utilizing modern nanotechnology to achieve targeted delivery to diseased myocardium is crucial for enhancing efficacy and reducing side effects. Third, insufficient evidence chain for clinical translation. The vast majority of current research remains at the preclinical stage. There is a lack of high-quality, large-sample randomized controlled trials to validate their efficacy and safety in humans. A dynamic monitoring system for ferroptosis-specific biomarkers (e.g., lipid peroxidation derivatives in plasma) in humans has not been established, hindering accurate efficacy assessment.

Looking ahead, future research on TCM against myocardial ferroptosis should advance in three directions: “Mechanism Elucidation”, “Technological Innovation”, and “Clinical Integration”. Mechanistically, cutting-edge technologies like single-cell sequencing, spatial transcriptomics, and organoids should be employed to reveal the heterogeneity of ferroptosis within the myocardial tissue microenvironment and its interaction with immune inflammation. Technologically, efforts should focus on developing targeted nano-drug delivery systems to improve the cardiac targeting and bioavailability of TCM components. In clinical translation, promoting evidence-based medical research and exploring advantageous integrated Chinese–Western medicine strategies—such as combining TCM with potential anti-ferroptosis properties with conventional chemotherapeutic drugs to enhance efficacy while reducing cardiotoxicity—is essential. In summary, viewing through the novel lens of ferroptosis and deeply exploring the multi-target regulatory wisdom of TCM will not only open new avenues for the prevention and treatment of cardiovascular diseases but also hold promise for promoting the interpretation and innovation of TCM theory within the context of modern life sciences.

## Figures and Tables

**Table 1:** Mechanism of action of Flavonoids regulating ferroptosis.

Composition Type	Composition Name	Source	Traditional Use	Experimental Model	Signaling Pathway	Mechanisms	Ref.
Flavonoids compounds	Baicalin	*Scutellaria baicalensis* Georgi	Clearing heat and drying dampness, purging fire and detoxification, hemostasis, tocolysis.	H9C2 cells (Rat cardiomyoblast cells)	SENP1/SIRT3	ROS↓, MDA↓, Iron concentration↓, GSH↑, GPX4↑, Xct↑	[127]
				DbCM male db/db mice			
	Wogonin			Mouse HL-1 cardiomyocytes	Alox15/15-HpETE	ALOX15↓, GPX4↑, 15-HpETE↓, MDA↓, 15-HETE↓, PTGS2↓, GSH/GSSG↓, 4-HNE↓	[128]
				SCM male and female adult C57BL/6 mice			
	Naringenin	Citrus grandis	Liqi Kuanzhong, dry dampness and phlegm, digestion and accumulation.	MI/RI male Sprague-Dawley (SD) rats	PI3K/AKT	MDA↓, SOD↑, Apoptosis index↓, Beclin-1↓, Bax↓, Bcl-2↑, p-AKT↑, cleaved caspase/caspase 3↓	[129]
				H9C2 cells	Nrf2/System xc-/GPX4	MPO↓, LPO↓, MDA↓, ROS↓, SOD↑, GSH↑, Fe2+↓, Nrf2↑, GPX4↑, SLC7A11↑, FTH1↑, FPN1↑, NOX1↓	[130]
				MI/RI SD rats			
				Embryonic rat cardiac cell line H9c2	p38MAPK	p-p38MAPK↑, ROS↓, cleaved caspase-3↓, MMP↑	[131]
	Isoliquiritigenin	*Glycyrrhiza uralensis* Fisch.	Tonifying spleen and replenishing qi, clearing heat and detoxifying, dispelling phlegm and relieving cough, relieving urgency and relieving pain, and harmonizing various medicines.	IIMI male C57BL6J mice	Nrf2/HO-1	MDA↓, ROS↓, SOD↑, GSH-Px↑, Nrf2↑, HO-1↑	[132]
				Neonatal mouse cardiomyocytes (NMCM)	Nrf2/HO-1/SLC7A11/GPX4	MDA↓, ROS↓, LDH↓, SOD↑, CAT↑, ACSL4↓, 4-HNE↓, GPX4↑, SLC7A11↑, Nrf2↑, HO-1↑	[133]
				I/R male C57BL6J mice			
	Licochalcone A			H9C2 cells	PI3K/AKT/MDM2/p53	ROS↓, Fe^2+^↓, MDA↓, SOD↑, GSSG↓, GSH↑, GSH/GSSG↑, p53↓, SLC7A11↑, GPX4↑, p-PI3K↑, p-AKT↑, p-MDM2↑	[134]
				DIC male ICR mice			
	Kaempferol	*Allium cepa* L., *Camellia sinensis* (L.) O. Kuntze. et al.	Antioxidant, anti-inflammatory, anti-tumor, cardiovascular protection.	H9c2 cells	Nrf2/SLC7A11/GPX4	LDH↓, SOD↓, GSH↓, GSH-Px↓, Fe^2+^↓, ROS↓, SLC7A11↑, GPX4↑, ACSL4↓, Nrf2↑, FTH1↑, FTL↑, MMP↑	[135]
	Galangin	*Alpinia officinarum* Hance	Warm stomach antiemetic, cold pain relief.	H9c2 cells	GSTP1/JNK	MDA↓, LDH↓, Ptgs2↓, GSH↑, GPX4↑, Nrf2↑, FPN↑, SLC7A11↑, GSTP1↑, p-JNK↓	[136]
				DIC male C57BL/6 mice			
	Total flavonoids from Clinopodium chinense	*Clinopodium chinense* (Benth).	Shufeng heat, detoxification swelling, cooling blood to stop bleeding.	H9c2 cells	MAPK, PI3K/AKT	MDA↓, LDH↓, SOD↑, CAT↑, GSH-Px↑, caspase-3↓, Bcl-2/Bax↓, p-ERK1/2↓, p-JNK↓, p-p38↓, PI3K↑, p-AKT↑, p53↓	[137]
				DIC male SD rats			
				H9c2 cells	AKT/Nrf2/HO-1	ROS↓, LDH↓, SOD↑, CAT↑, GSH-Px↑, POD↑, HO-1↑, p-AKT↑, Nrf2↑	[138]
				I/R male SD rats			
	tournefolic acid B			H9c2 cells	PI3K/AKT	LDH↓, Caspase-3↓, Caspase-9↓, ROS↓, MDA↓, SOD↑, CAT↑, GSH-Px↑, p-PI3K↑, p-AKT↑, Bcl-2/Bax↑, p-JNK↑	[139]
				MI/RI male SD rats			

Note: “↑” represent upward adjustments, “↓” represent downward adjustments.

**Table 2:** Mechanism of action of Glycosides regulating ferroptosis.

Composition Type	Composition Name	Source	Traditional Use	Experimental Model	Signaling Pathway	Mechanisms	Ref.
Glycosides compounds	Ginsenoside Rg3	*Panax ginseng* C. A. Mey.	Tonifying vital qi, restoring pulse, tonifying spleen and lung, nourishing body fluid and blood, tranquilizing mind and benefiting intelligence.	H9C2 cells	FoxO3a	Sirt1↑, PGC-1α↑, Nrf1↑, Nrf2↑, HO-1↑, SOD1↑, Bax↓, Bcl-2↑	[140]
				MI/RI SD rats			
				H9C2 cells	keap1/Nrf2/GPX4	Fe^2+^↓, GSH↑, GPX4↑, FTH1↑, Nrf2↑, HO-1↑, NQO1↑, Keap1↓	[141]
				MI/RI male C57BL/6 mice			
	20(R)-ginsenoside Rg3			PC12 cells	Nrf2/HO-1	Nrf2↑, HO-1↑, ROS↓, MDA↓, SOD↑, MMP↑	[142]
				I/R male SD rats			
	Ginsenoside Rg1			DIC male wild-type C57BL/6J mice	ERS, Autophagy	LC3B/LC3A↓, p62↑, ATG5↓, Beclin-1↓, p-P70S6K↑, JNK1↓	[143]
				DbCM Wistar rats	ERS-induced apoptosis	GRP78↓, Caspase-12↓, CHOP↓	[144]
	Ginsenoside Rb1			DIC male C57BL/6 mice	AMPK/mTOR, GPX4/FTH1	SOD↑, GSH↑, MDA↓, p62↓, LC3-II↓, GPX4↑, FTH1↑, Nrf2↑	[146]
	Diosgenin	*Dioscorea nipponica* Makino	Qufeng dehumidification, Shujin Tongluo, Huoxue Zhitong, cough and asthma.	H9C2 cells	Nrf2/Sirt2	ROS↓, MDA↓, SOD↑, GSH↑, GSH-Px↑, Nrf2↑, Keap1↓, HO-1↑, NQO1↑, Sirt2↑, FOXO3a↑	[147]
				DIC male-SD rats			
				Mouse myocardial cell line (HL-1)	PDK1/AKT/mTOR	Cleaved Caspase-3↓, Bcl-2↑, Beclin1↓, Atg5↓, MDA↓, SOD↑, p-PDK1↑, p-AKT↑, p-mTOR↑	[148]
				H9C2 cells	Nrf2-GPX4	4-HNE↓, MDA↓, SOD↑, CAT↑, GSH↑, GSH-Px↑, GPX4↑, ACSL4↓, Fe^2+^↓, ROS↓, Nrf2↑, HO-1↑, TfR1↓, DMT1↓, FTH1↑, FTL↑, FPN↑	[149]
				DIC male SD rats			
				H9C2 cells	PERK-eIF2α-ATF4	MDA↓, ROS↓, SOD↑, GSH↑, TFRC↓, PTGS2↓, SLC7A11↑, GPX4↑, Fe^2+^↓, LDH↓, p-PERK↓, p-eIF2α↓, ATF4↓, GRP78/BiP↓	[150]
				MI male C57BL/6 mice			
	Salidroside	*Rhodiola crenulata* (Hook. f. et Thoms.) H. Ohba	Tonifying Qi and activating blood circulation, Tongmai asthma.	H9C2 cells	AMPK	ROS↓, MDA↓, GSH↑, GPX4↑, 4-HNE↓, p-AMPK↑, p-ACC↑	[151]
				DIC male C57/BL mice			
	Astragaloside IV	*Astragalus membranaceus* (Fisch.) Bge.	Tonifying qi and ascending yang, consolidating the surface and stopping sweating, promoting diuresis and detumescence, generating body fluid and nourishing blood, promoting stagnation and dredging arthralgia, supporting toxin and expelling pus, astringing sores and promoting granulation.	H9C2 cells	CD36/ACSL4/GPX4	CD36↓, Fe^2+^↓, ROS↓, MDA↓, ACSL4↓, p53↓, GPX4↑	[152]
				DbCM male SD rats			
				DIC SD male rats	Nrf2	SOD↑, CAT↑, GPx4↑, MDA↓, NOX2↓, NOX4↓, Nrf2↑, Keap1↓	[153]
	Pedunculoside	Ilex genus	Anti-inflammatory, anti-oxidation, anti-tumor	High glucose-induced H9c2 cells	Nrf2/HO-1	Nrf2↑, HO-1↑, NQO1↑, SOD↑, MDA↓	[154]
	Sarmentosin	*Sedum sarmentosum* Bunge	Promotes diuresis and alleviates jaundice; clears heat and detoxifies.	H9C2 cells	p62-Keap1-Nrf2	MDA↓, GSH↑, SOD↑, GPX4↑, PTGS2↓, Nrf2↑, HO-1↑, NQO-1↑, Keap1↓, p62↓	[155]
				DIC male C57BL/6 mice			
	Panaxatriol Saponin	*Panax notoginseng* (Burk.) F. H. Chen	Disperse blood stasis and stop bleeding; reduce swelling and relieve pain.	H9C2 cells	Keap1/Nrf2	Keap1↓, Nrf2↑, HO-1↑, NQO1↑, SOD1↑, SOD2↑, ROS↓	[156]
				MI/RI male SD rats			

Note: “↑” represent upward adjustments, “↓” represent downward adjustments.

**Table 3:** Mechanism of action of Phenolic compounds regulating ferroptosis.

Composition Type	Composition Name	Source	Traditional Use	Experimental Model	Signaling Pathway	Mechanisms	Ref.
Phenolic compounds	Yellow wine polyphenol compounds	Rice wine	Warm the middle burner and dispel cold, invigorate blood circulation and unblock meridians, tonify qi and blood, relax tendons and activate meridians, direct herbs to their meridian pathways, calm the spirit and aid sleep.	H9C2 cells	Nrf2	ROS↓, MDA↓, SOD↑, Catalase↑, HO-1↑, NQO-1↑, GCLM↑, Nrf2↑, Bax↓, Bcl-2↑	[157]
				DIC-male SD rats			
	Astragalus polyphenols	*Astragalus membranaceus* (Fisch.) Bge.	Tonifying qi and ascending yang, consolidating the surface and stopping sweating, promoting diuresis and detumescence, generating body fluid and nourishing blood, promoting stagnation and dredging arthralgia, supporting toxin and expelling pus, astringing sores and promoting granulation.	H9c2 cells and human myocardial cells (AC16)	PI3K/AKT/Nrf2	ANP↓, BNP↓, ROS↓, MMP↑, p-PI3k↑, p-AKT↑, Nrf2↑, HO-1↑, GCLC↑, GPX4↑, FTH1↑	[158]
	Resveratrol	*Polygonum cuspidatum* Sieb. et Zucc.	Promotes diuresis and alleviates jaundice, clears heat and detoxifies, disperses blood stasis and relieves pain, suppresses cough and resolves phlegm.	H9C2 cells	MAPK	GSH↑, ROS↓, GPX4↑, PTGS2↓, ACSL4↓, NCOA4↓, Fe^2+^↓, p-ERK/ERK↓, p-JNK/JNK↓, p-p38/p38↓	[159]
				DIC male C57/BL mice			
				H9C2 cells	p62-Nrf2/HO-1	MDA↓, ROS↓, MMP↑, p62↑, Nrf2↑, HO-1↑, GPX4↑, GSH↑	[160]
				DIC male C57/BL mice			
				MI/RI diabetic male SD rats	AMPK/p38/Nrf2	SOD↑, GSH↑, ROS↓, p-AMPK↑, p-p38↓ Nrf2↑, HO-1↑	[161]
	Caffeic acid phenethyl ester	Coffea arabica	anti-cancer, antioxidant, anti-inflammatory and immunoregulation	H9c2 cells and AC16 cells	ROS-MLKL	ROS↓, GSH↑ RIPK1↓, RIPK3↓, p-MLKL↓	[162]
				DIC male BALB/c mice			
	6-Gingerole	*Zingiber officinale* Rosc.	Relieves exterior symptoms and dispels cold; warms the middle and stops vomiting; transforms phlegm and relieves cough; neutralizes fish and crab toxins.	H9c2 cells	Nrf2/HO-1	Nrf2↑, HO-1↑, SOD↑, MDA↓, FACL4↓, GPX4↑	[163]
				DbCM male C57BL/6 mice			
	Punicalagin	*Punica granatum* L.	Astringent to the intestines, stops diarrhea, stops bleeding, expels parasites.	H9c2 cells	Nrf2/HO-1	Nrf2↑, HO-1↑, ROS↓, MMP↑, Bax↓, Bcl-2↑	[164]
	Curcumin	*Curcuma longa* L.	Promotes blood circulation and qi flow, unblocks meridians and alleviates pain.	H9c2 cells	Nrf2/HO-1	Nrf2↑, HO-1↑, ROS↓, ACSL4↓, COX1↓, GPX4↑	[73]
				DbCM male New Zealand rabbits			
				H9c2 cells	AKT/Nrf2/ARE	ROS↓, MMP↑, p-AKT↑, Nrf2↑, HO-1↑, GCLC↑, NLRP3↓, caspase-1↓	[165]
				DbCM male SD rats			
				H9c2 cells	Nrf2/HO-1	Nrf2↑, HO-1↑, SOD↑, GSH-Px↑, ROS↓, MMP↑, Bax↓, Bcl-2↑	[166]
				DbCM SD rats			

Note: “↑” represent upward adjustments, “↓” represent downward adjustments.

**Table 4:** Mechanism of action of Polysaccharides regulating ferroptosis.

Composition Type	Composition Name	Source	Traditional Use	Experimental Model	Signaling Pathway	Mechanisms	Ref.
Polysaccharide compounds	Fucoidan	Phaeophyceae	Softens hardened masses and disperses nodules, Resolves phlegm and promotes diuresis, Clears heat.	The HL-1 mouse cardiomyocyte cell line	Nrf2/GPX4	ROS↓, MDA↓, Nrf2↑, HO-1↑, GPX4↑, TfR1↑, FTH1↑, Ptgs2↓	[168]
				DIC male C57BL/6 mice			
	Ophiopogon japonicus polysaccharide	*Ophiopogon japonicus* (L. f.) Ker-Gawl.	Nourish yin and generate fluids, Moisten the lungs and clear the heart.	AC16 cells	Nrf2/GPX4	Nrf2↑, GPX4↑, MDA↓, ROS↓, TfR1↓, Fe^2+^↓, MMP↑	[169]
	Astragalus polysaccharides	*Astragalus membranaceus* (Fisch.) Bge.	Tonifying qi and ascending yang, consolidating the surface and stopping sweating, promoting diuresis and detumescence, generating body fluid and nourishing blood, promoting stagnation and dredging arthralgia, supporting toxin and expelling pus, astringing sores and promoting granulation.	H9c2 cells	AMPK/mTOR	p-AMPK↓, p-mTOR↑, p62↓, Cleaved Caspase-3↓, Bcl-2↑	[171]
				DIC male C57BL/6 mice			
	Lycium barbarum polysaccharide	*Lycium barbarum* L.	Nourishes the liver and kidneys, enriches essence, and improves eyesight.	H9c2 cells	Nrf2	LDH↓, CK↓, MDA↓, SOD↑, Nrf2↑, p62↓	[172]
				I/R male SD rats,			

Note:“↑” represent upward adjustments, “↓” represent downward adjustments.

**Table 5:** Mechanism of action of Terpenoids regulating ferroptosis.

Composition Type	Composition Name	Source	Traditional Use	Experimental Model	Signaling Pathway	Mechanisms	Ref.
Terpenoid compounds	Salvia miltiorrhiza (Dan Shen)	*Salvia miltiorrhiza* Bge.	Promotes blood circulation and removes blood stasis; Regulates menstruation and alleviates pain; Calm the mind and dispel restlessness; Cool the blood and eliminate abscesses.	H9c2 cells	Nrf2	ROS↓, MDA↓, Nrf2↑, Xct↑, GPX4↑, GSH↑, Fe^2+^↓	[173]
				MI male C57BL/6 mice			
	Tanshinone I			H9c2 cells	Nrf2	p-AKT↑, Nrf2↑, HO-1↑, NQO1↑, ROS↓, MDA↓, SOD↑, GSH-Px↑, Bcl-2↑, Bax↓	[174]
				DIC male C57BL/6 mice			
	Tanshinone IIA			AC16 cells	Nrf2-xCT/Gpx4/HO-1	HDAC1↓, Nrf2↑, Xct↑, GPX4↑, HO-1↑, ROS↓, MDA↓, Fe^2+^↓, GSH↑	[175]
				MI/RI SD rats			
	Dihydroartemisinin	*Artemisia annua* L.	Clears deficiency heat, eliminates bone steaming, relieves summer heat, stops malaria, and reduces jaundice.	H9c2 cells	Nrf2	ATG5↑, LC3B-II↑, p62↓, keap1↓, Nrf2↑, HO-1↑, NQO1↑, ROS↓, MDA↓, SOD↑, GSH↑, GPX4↑, PTGS2↓	[176]
				DIC male or female C57BL/6 mice			
				arsenic-induced myocardial injury male SD rats	modulating oxidative stress and inflammatory responses	SOD↑, GSH↑, TNF-α↓, IL-6↓, LDH↓	[177]

Note: “↑” represent upward adjustments, “↓” represent downward adjustments.

**Table 6:** Mechanism of action of Lignan compounds regulating ferroptosis.

Composition Type	Composition Name	Source	Traditional Use	Experimental Model	Signaling Pathway	Mechanisms	Ref.
Lignan compounds	Schisandrol B	*Schisandra chinensis* (Turcz.) Baill.	Converge solid astringent, replenishing qi and promoting fluid, tonifying kidney and calming heart.	H9c2 cells	SLC7A11/GPX4	ROS↓, MDA↓, p53↓ SLC7A11↑, GPX4↑, GSH↑, Fe^2+^↓	[178]
				DbCM male C57BL/6 J mice			
	Protosappanin A	*Caesalpinia sappan* L.	Blood stasis, swelling and pain.	H9c2 and HEK-293 cells	ACSL4/FTH1	ROS↓, MDA↓, GSH↑, GPX4↑, PTGS2↓, ACSL4↓, FTH1↑, Fe^2+^↓	[179]
				DIC male C57BL/6 mice			
	Honokiol	*Magnolia officinalis* Rehd. et Wils.	Drying dampness and eliminating phlegm, removing fullness.	H9c2 cells	SIRT1-Nrf2	SIRT1↑, Nrf2↑, HO-1↑, NQO-1↑, SOD↑, ROS↓, MDA↓, Bcl-2/Bax↑, Caspase-3↓	[180]
				MI/RI SD rats			

Note: “↑” represent upward adjustments, “↓” represent downward adjustments.

**Table 7:** Mechanism of action of Aldehyde compounds regulating ferroptosis.

Composition Type	Composition Name	Source	Traditional Use	Experimental Model	Signaling Pathway	Mechanisms	Ref.
Aldehyde compounds	Cinnamaldehyde	*Cinnamomum cassia* Presl.	Reinforcing fire to help Yang, inducing fire to return to Yuan, dispersing cold and relieving pain, warming and dredging meridians.	H9c2 cells	Nrf2/HO-1	Nrf2↑, HO-1↑, ROS↓, MDA↓, SOD↑, GSH↑, GSH-Px↑, GPX4↑, ACSL4↓, PTGS2↓	[181]
				DIC male SD rats			

Note: “↑” represent upward adjustments, “↓” represent downward adjustments.

**Table 8:** Mechanism of action of Chinese Herbal Remedies regulating ferroptosis.

Chinese Herbal Remedy	Composition	Experimental Model	Signaling Pathway	Mechanisms	Ref.
Guanxinning injection	Phenolic acids (salvianolic acid B, danshensu, protocatechuic aldehyde), diterpene quinones (tanshinone IIA, cryptotanshinone), alkaloids (ligustrazine, ferulic acid)	CHF C57BL/6 mice	SLC7A11/GPX4	SLC7A11↑, GPX4↑, FTH1↑	[183]
YiQi FuMai injection	Saponins (ginsenosides, ophiopogonin D), lignans (schisandrin)	H9c2 cells	xCT/GPX4	SLC7A11↑, SLC3A2↑, GPX4↑, GSH↑, Total iron↓, ROS ↓, MDA↓, 4-HNE↓	[184]
		SCM male SD rats			
Qishen granule	Saponins (Astragaloside I, Astragaloside III, Isoflavone Glucoside), Phenolic acids (Tannic acid B, Chlorogenic acid), quinones (cryptotanshinone, tanshinone IIA, dihydrotanshinone I), flavonoids (quercetin and its derivatives, kaempferol, luteolin), organic acids, amino acids, alkaloids, lignans, coumarins	H9c2 cells	SIRT3	ROS↓, MDA↓, SOD↑, Bax↑, Bcl-2↑, Bcl-2/Bax↑, SIRT3↑	[188]
		DIC C57BL/6 mice			
		H9c2 cells	Nrf2	ROS↓, MDA↓, GSH↑, GPX4↑, Nrf2↑, FPN↑, FTH1↑, FTMT↑	[189]
		DIC male C57 BL/6 mice			
Zhilong Huoxue Tongyu capsule	Saponin (astragaloside), polysaccharide (astragalus polysaccharide), flavonoid (kaempferol, quercetin)	MI/RI C57BL/6J mice	PI3K/AKT/Nrf2	p-PI3K↑, p-AKT↑, Nrf2↑, HO-1↑, GPX4↑, ROS ↓, MDA↓, SOD↑, GSH↑, Fe^2+^↓, ACSL4↓, GPX4↑	[194]
QiShenYiQi dripping pill	Triterpene saponins (astragaloside), diterpene quinones (tanshinone IIA, cryptotanshinone), saponins (notoginsenoside R1, ginsenoside), volatile oil (trans-nerolidol).	H9C2 cells	SLC7A11/GPX4	MDA↓, 4-HNE↓, ACSL4↓, FHC↓, GSH↑, GPX4↑, SLC7A11↑, Nrf1↑	[195]
		ICR male mice			
Ling-Gui-Zhu-Gan decoction	Triterpenoid acids (pachymic acid, dehydropachymic acid), volatile oils (cinnamaldehyde, cinnamic acid), sesquiterpene lactones (atractylenolide), flavonoids (glycyrrhizic acid, glycyrrhetinic acid).	H9C2 cells	Nrf2	Nrf2↑, GPX4↑, Fpn↑, Ptgs2↓	[196]
		DIC male SD rats			
HJ11 decoction	Phenolic acids (chlorogenic acid), iridoids (harpagoside, geniposidic acid), quinones (tanshinone IIA, salvianolic acid B), anthraquinones (resveratrol, emodin), volatile oils (cinnamaldehyde), flavonoids (glycyrrhizic acid, isoliquiritigenin), phthalide (ligustilide).	H9C2 cells	ACSL4	FTH1↑, GPX4↑, ACSL4↓, COX2↓	[197]
		I/R male SD rats			

Note: “↑” represent upward adjustments, “↓” represent downward adjustments.

## Data Availability

No new data were created or analyzed in this study. Data sharing is not applicable to this article.

## Abbreviations

The following abbreviations are used in this manuscript:

HCM	Hypertrophic cardiomyopathy
DCM	Dilated cardiomyopathy
DbCM	Diabetic cardiomyopathy
ICM	Ischemic Cardiomyopathy
MI/RI	Myocardial Ischemia–Reperfusion Injury
DOX	Doxorubicin
DIC	Doxorubicin-induced cardiotoxicity
SCM	Septic cardiomyopathy
GPX4	Glutathione peroxidase 4
TCM	Traditional Chinese Medicine
LOXs	Lipoxygenases
GSH	Glutathione
LPO	Lipid peroxides
LOH	Lipid alcohols
H_2_O_2_	Hydrogen peroxide
Tf	Transferrin
TFR1	Transferrin receptor 1
Fe^2+^	Ferrous ions
NCOA4	Nuclear receptor coactivator 4
UFA	Unsaturated fatty acids
PUFA	Polyunsaturated fatty acids
ACSL4	Acyl-CoA synthetase long-chain family member 4
AA	Arachidonic acid
AdA	Adrenic acid
CoA	Coenzyme A
LPCAT3	Lysophosphatidylcholine acyltransferase 3
PUFA-PL	PUFA-containing phospholipids
LOOH	Lipid hydroperoxides
Nrf2	Nuclear factor erythroid 2-related factor 2
HO-1	Heme oxygenase-1
NQO1	NAD(P) H quinone dehydrogenase 1
FSP1	Ferroptosis suppressor protein 1
LTCC	L-type calcium channel
NTBI	Non-transferrin-bound iron
ERS	Endoplasmic reticulum stress
STZ	Streptozotocin
HG	High glucose
T1DM	Type 1 diabetes mellitus
HF	Heart failure
ACOT1	Acyl-CoA thioesterase 1
Fer-1	Ferrostatin-1
HMGB1	High mobility group box 1
DXZ	Dexrazoxane
SOD	Superoxide dismutase
GSTP1	Glutathione S-transferase P1
LPS	Lipopolysaccharide
STING	Stimulator of interferon genes
MDA	Malondialdehyde
CHF	Chronic heart failure
